# Immunosenescence and cytomegalovirus-associated immune signatures on severe acute respiratory syndrome coronavirus 2 booster responses

**DOI:** 10.1093/gerona/glag095

**Published:** 2026-04-17

**Authors:** Irene Reina-Alfonso, Pablo Álvarez-Heredia, Isabel M Vallejo-Bermúdez, Ana Navas-Romo, Mónica Espinar-García, Fakhri Hassouneh, Ana Belén Pérez, Raquel Tarazona, Rafael Solana, Alexander Batista-Duharte, Juan Molina, Alejandra Pera

**Affiliations:** Molecular Immunology Group (CTS-208), Department of Cell Biology, Physiology and Immunology, University of Córdoba, Córdoba, Spain; Immunology and Allergy Group (GC01), Maimónides Biomedical Research Institute of Córdoba (IMIBIC), University of Córdoba, Reina Sofia University Hospital, Córdoba, Spain; Immunology and Allergy Group (GC01), Maimónides Biomedical Research Institute of Córdoba (IMIBIC), University of Córdoba, Reina Sofia University Hospital, Córdoba, Spain; Anatomy Pathology Service, Hospital Nostra Senyora de Meritxell, Andorra; Molecular Immunology Group (CTS-208), Department of Cell Biology, Physiology and Immunology, University of Córdoba, Córdoba, Spain; Immunology and Allergy Group (GC01), Maimónides Biomedical Research Institute of Córdoba (IMIBIC), University of Córdoba, Reina Sofia University Hospital, Córdoba, Spain; Immunology and Allergy Group (GC01), Maimónides Biomedical Research Institute of Córdoba (IMIBIC), University of Córdoba, Reina Sofia University Hospital, Córdoba, Spain; Immunology and Allergy Service, Reina Sofia University Hospital, Córdoba, Spain; Molecular Immunology Group (CTS-208), Department of Cell Biology, Physiology and Immunology, University of Córdoba, Córdoba, Spain; Immunology and Allergy Group (GC01), Maimónides Biomedical Research Institute of Córdoba (IMIBIC), University of Córdoba, Reina Sofia University Hospital, Córdoba, Spain; Immunology and Allergy Group (GC01), Maimónides Biomedical Research Institute of Córdoba (IMIBIC), University of Córdoba, Reina Sofia University Hospital, Córdoba, Spain; Microbiology Unit, Reina Sofía University Hospital, Córdoba, Spain; Clinical and Molecular Microbiology Group (GC24), Maimónides Biomedical Research Institute of Córdoba (IMIBIC), Reina Sofia University Hospital, University of Córdoba, Córdoba, Spain; Centro de Investigación Biomédica en Red de Enfermedades Infecciosas (CIBERINFEC), Spain; Immunology Unit, Department of Physiology, University of Extremadura, Cáceres, Spain; Molecular Immunology Group (CTS-208), Department of Cell Biology, Physiology and Immunology, University of Córdoba, Córdoba, Spain; Immunology and Allergy Group (GC01), Maimónides Biomedical Research Institute of Córdoba (IMIBIC), University of Córdoba, Reina Sofia University Hospital, Córdoba, Spain; Immunology and Allergy Service, Reina Sofia University Hospital, Córdoba, Spain; Immunology and Allergy Group (GC01), Maimónides Biomedical Research Institute of Córdoba (IMIBIC), University of Córdoba, Reina Sofia University Hospital, Córdoba, Spain; Immunology and Allergy Group (GC01), Maimónides Biomedical Research Institute of Córdoba (IMIBIC), University of Córdoba, Reina Sofia University Hospital, Córdoba, Spain; Immunology and Allergy Service, Reina Sofia University Hospital, Córdoba, Spain; Molecular Immunology Group (CTS-208), Department of Cell Biology, Physiology and Immunology, University of Córdoba, Córdoba, Spain; Immunology and Allergy Group (GC01), Maimónides Biomedical Research Institute of Córdoba (IMIBIC), University of Córdoba, Reina Sofia University Hospital, Córdoba, Spain; (Biological Sciences Section)

**Keywords:** BNT162b2, severe acute respiratory syndrome coronavirus 2, hybrid immunity, cytomegalovirus infection, immunosenescence

## Abstract

Aging remodels antiviral immunity, yet its influence on responses to repeated mRNA vaccination is not fully defined. We evaluated humoral and severe acute respiratory syndrome coronavirus 2 (SARS-CoV-2) spike–specific T-cell responses in 41 adults—stratified by age (<50 vs. ≥60 years), sex, prior SARS-CoV-2 infection, and cytomegalovirus (CMV) serostatus—before and after a fourth dose of the bivalent BNT162b2 vaccine. Anti-RBD IgG titers increased in nearly all participants, with no measurable impact of age, sex, infection history, or CMV status, and baseline titers predicted post-booster antibody levels. In contrast, cellular immunity showed clear heterogeneity across aging-related variables. Although the booster enhanced IFN-γ production and reduced TNF-α-associated inflammatory activity at the cohort level, older adults and males exhibited significantly lower post-boost frequencies of IFN-γ–producing CD4+ T cells. Prior SARS-CoV-2 infection was associated with attenuated CD4+ recall responses, whereas infection-naïve and female participants showed the strongest functional gains. Immunosenescence markers were associated with reduced cellular responsiveness. CMV-related immune remodeling—including higher anti-CMV IgG levels and expansions of differentiated CD8+ subsets—correlated with diminished IFN-γ responses in CD4+ and CD8+ T cells after boosting, suggesting that chronic CMV imprinting constrains heterologous antiviral immunity even in mid-adult life. Humoral and cellular changes were largely uncoupled, supporting the need to evaluate both arms of adaptive immunity. These findings indicate that while a fourth bivalent BNT162b2 dose reliably reinforces humoral immunity across ages, the magnitude and quality of cellular responses are shaped by age, sex, infection history, and CMV-associated immunosenescence. Incorporating immune-aging markers into vaccination strategies may improve booster efficacy in older populations.

## Graphical Abstract

**Graphical Abstract:**
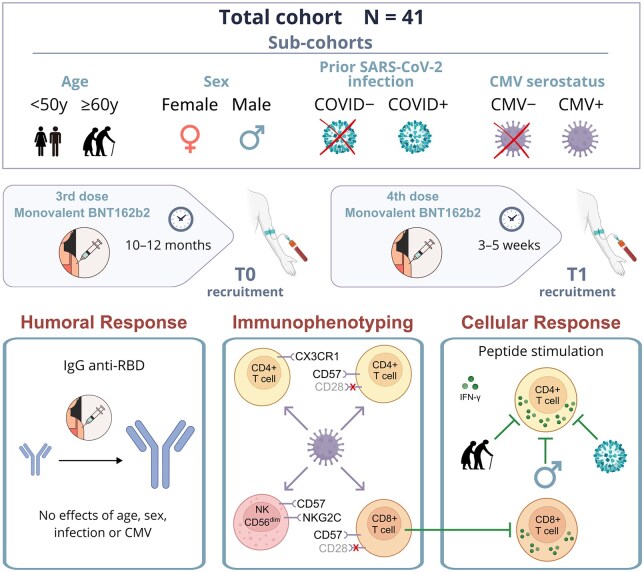

## Introduction

The development of mRNA vaccines against severe acute respiratory syndrome coronavirus 2 (SARS-CoV-2) has had a major impact on global morbidity and mortality, particularly during the initial waves of the COVID-19 pandemic. These vaccines demonstrated high efficacy in preventing severe illness and death, leading to mass immunization campaigns worldwide. Early evidence also suggested that vaccination may further reduce systemic inflammation during acute infection and recovery,^1^ potentially contributing to the observed reduction in COVID-19 severity and mortality. However, with time, waning immunity—whether from natural infection or vaccination—coupled with the emergence of immune-evasive variants such as Omicron, prompted the implementation of booster strategies aimed at restoring protection.^2^ Booster recommendations were initially restricted to high-risk populations and later extended to the general public.^2^ As in other countries, at-risk groups in Andalusia (Spain) include individuals over 60 years of age, people with chronic illnesses or immunocompromised conditions, and health and social care workers, among others. In this region, Pfizer-BioNTech (BNT162b2) mRNA vaccine has been the predominant COVID-19 vaccine administered throughout all phases of the vaccination campaign. While the initial 3 doses of the vaccine targeted the ancestral Wuhan SARS-CoV-2 strain (monovalent), subsequent booster doses have been reformulated to align with the most prevalent variants circulating at the time of each campaign. Specifically, the fourth dose consisted of a bivalent formulation including both the original strain and Omicron BA.4/BA.5, whereas the most recent campaigns have used monovalent boosters targeting Omicron variants XBB.1.5, JN.1, and KP.2.^3^ These decisions were largely based on declining neutralizing antibody titers and increasing rates of breakthrough infection, while the role of cellular immunity in sustaining long-term protection remained insufficiently addressed.^4^

The rationale for additional doses has been supported by modeling studies predicting that mRNA boosters, particularly when combined with prior infection, would offer the highest levels of protection against severe outcomes and reinfection.^5^ The model also revealed a decline in protection against symptomatic infection and transmission, especially with the emergence of Omicron subvariants, underscoring the need for variant-adapted strategies.^2^ Wachter et al.^6^ studied the effects of a BA.4/BA.5 bivalent mRNA booster in individuals with or without prior SARS-CoV-2 infection, with at least 3 prior mRNA doses. Although both groups mounted strong humoral responses, only infection-naïve individuals exhibited a significant post-boost expansion of Omicron-specific memory B cells and CD4+ T cells. Conversely, features such as IgA+CXCR3+ switched memory B cells were restricted to previously infected subjects, reflecting the impact of hybrid immunity on mucosal imprinting.^6^

Some studies have raised questions about the possibility of T cell tolerance or exhaustion after successive vaccine administrations. It has been suggested that repeated mRNA vaccination may induce class-switching toward IgG4, potentially limiting Fc-mediated effector functions.^7^ In a study with mice, Gao et al.^8^ observed evidence of tolerance and immunological exhaustion in mice exposed to multiple doses of the vaccine. They observed from the fourth inoculation onward, a decrease in antibodies, a loss of effectiveness of neutralizing antibodies, a decrease in CD4+ and CD8+ T-cell responses, as well as an increase in PD-1 or LAG-3.^8^ However, Saiag et al.^9^ reported that in infection-naïve adults aged ≥60, a fourth BNT162b2 dose elicited robust IgG and CD4+ T-cell responses, with no evidence of anergy or immunosenescence. In older adults, hybrid immunity promotes a qualitatively superior humoral profile. In particular, vaccinated individuals with prior infection displayed increased frequencies of polyfunctional CD4+ and CD8+ T cells expressing IFN-γ, TNF-α, and granzyme B,^10^ supporting the idea that infection followed by vaccination may provide stronger cellular training than the reverse. Additionally, a recent meta-analysis^11^ confirmed that heterologous booster regimens, particularly those involving mRNA platforms, outperform homologous strategies in terms of neutralizing antibody titers across wildtype and variant strains, with no evidence of reduced immunogenicity. Moreover, recent studies have found no evidence of immune exhaustion after repeated SARS-CoV-2 vaccination in both vulnerable and healthy populations.^12^ These findings challenge the notion that repeated boosting might lead to functional exhaustion and instead support a flexible, adaptive approach.

Nevertheless, it has been shown that individuals with prior ancestral infection followed by Omicron reinfection displayed poor humoral boosting after a subsequent vaccine dose, whereas those initially infected with Omicron had the highest antibody titers,^13^ underscoring the importance of immune imprinting. A similar pattern emerged in a separate cohort where third doses enhanced anti-spike IgG but failed to significantly increase IFN-γ production in IGRA assays,^14^ raising questions about the sensitivity of conventional measures to capture meaningful cellular activity.

Beyond hybrid status, age remains a critical modifier of immune responses. Palacios-Pedrero et al.^15^ demonstrated that, compared with young individuals, older adults exhibit reduced neutralizing antibody titers, decreased memory B cell frequencies, and diminished polyfunctionality in CD8+ T cells, partly explained by a loss of naïve T cells and accumulation of senescent phenotypes. Sex-related differences in vaccine responses have been reported, with female individuals generally developing higher antibody levels than males.^16^ However, longitudinal data suggest that booster doses may attenuate these immune disparities over time.^17^ In nursing home residents, although initial responses differed by sex among previously infected individuals, booster doses equalized humoral responses across sexes and to Omicron variants.^17^

Another well-known modulator of the immune system is latent cytomegalovirus (CMV) infection, which is now recognized as an early driver of immunosenescence.^18^ Its impact on vaccine-induced immune responses has been widely discussed in the literature.^19^ In this sense, some authors have reported little to no differences in COVID-19 vaccine responses based on CMV serostatus shortly after vaccination.^18^ However, other data show that CMV-seropositive individuals tend to exhibit a decline in humoral response several months post-vaccination, especially among older individuals,^20^ supporting the need for periodic booster doses in this population.

Considering these findings, it is evident that repeated mRNA vaccination interacts with prior infection, and that age, sex, and coinfection with other viral agents will contribute to a complex immunological landscape that is not yet fully understood. Although parallel analysis of humoral immunity together with functional and phenotypic characterization of antigen-specific T-cell responses has been addressed in other studies, few have done so under combined stratification by age, sex, CMV serostatus, and prior SARS-CoV-2 infection. Furthermore, relatively few studies have performed an in-depth characterization of these responses after a fourth dose of a bivalent mRNA vaccine in infection-naïve individuals, particularly with CMV-stratified analyses, despite the known impact of CMV on T-cells phenotype and functionality.^21^

To address these knowledge gaps, we evaluated humoral and cellular responses to a fourth dose of the bivalent BNT162b2 vaccine in a well-characterized adult cohort. All participants had received 3 prior monovalent BNT162b2 doses and were either SARS-CoV-2 naïve or had experienced infection at least 5 months earlier—following recommendations,^22^—after at least one vaccine dose. The cohort included individuals under 50 and over 60 years of age, of both sexes, and stratified by CMV serostatus. We analyzed the humoral and cellular responses, including IFN-γ and TNF-α production by CD4+ and CD8+ T cells alongside detailed phenotyping, aiming to provide new insights into the immunological landscape after repeated mRNA vaccination, and to address key knowledge gaps regarding how aging and latent viral coinfections modulate the booster response.

## Methods

### Study design and participants

To achieve this goal, blood samples were collected from 41 healthy donors 10-12 months after the third dose of the monovalent vaccine BNT162b2 (T0) and 3-5 weeks after the bivalent booster (fourth dose) administration (T1) (Figure S1). The research involved volunteers from southern Spain, recruited between September 2022 and January 2023.

Several sub-cohorts were established according to age, sex, SARS-CoV-2 previous infection, or CMV serostatus (Table S1). For the Age sub-cohort, donors were divided into younger than 50 years old (<50) and 60 years old or older (≥60). All individuals previously infected by SARS-CoV-2 (COVID+) had mild symptoms or were asymptomatic with a positive antigen test result. Following the vaccination recommendations at that time, the SARS-CoV-2 infection must have occurred at least 5 months before the booster dose. Except for one, all COVID+ individuals had received a minimum of 2 doses of the vaccine prior to their initial SARS-CoV-2 infection.

### Ethics

The study was approved by the Ethics Committee of Reina Sofía University Hospital of Córdoba (Spain) (Identification of risk biomarkers associated with the immune response against SARS-CoV-2 infection [committee reference no. 5094]). Inclusion criteria: (a) no history of prior contact with SARS-CoV-2 and no COVID-19 symptoms for non-infected individuals or (b) a past infection occurring more than 5 months ago for infected individuals. For all, no history of cardiovascular disease, cancer, or autoimmune disorders, and the absence of ongoing immunosuppressive treatments. All participants read and signed the informed consent.

### SARS-CoV-2 IgG anti-RBD protein and anti-CMV IgG determination

Anti–SARS-CoV-2 Spike receptor-binding domain (RBD) IgG levels were quantified by ELISA using recombinant RBD-coated plates and a standard curve for absolute antibody concentration. Serum samples were analyzed in duplicate, and results were expressed as ng/mL after blank subtraction and dilution-factor adjustment.

Anti-CMV IgG and IgM antibodies were measured by chemiluminescent immunoassay (CLIA) using the Liaison XL platform (DiaSorin) at the Microbiology Service of the Reina Sofía University Hospital.

Detailed protocols are provided in Methods S1 (Supplementary Material).

### Functionality assay

Peripheral blood mononuclear cells (PBMCs) were isolated by density-gradient centrifugation and stimulated with a SARS-CoV-2 Spike peptide pool. After antigenic stimulation in the presence of brefeldin A, cells were stained for viability, surface markers, and intracellular cytokines to quantify IFN-γ and TNF-α production by CD4+ and CD8+ T cells. Detailed information on reagents, peptide concentrations, incubation conditions, and antibody panels is provided in Methods S2 and Table S2.

### Immune cells immunophenotyping

Fresh whole blood was stained with monoclonal antibody panels for phenotyping of T cells and innate immune subsets, followed by red-cell lysis and acquisition by flow cytometry. Memory T-cell subsets were defined as follows: effector memory T cells (TEM): CD45RA−CCR7−; terminally differentiated effector memory T cells re-expressing CD45RA (TEMRA): CD45RA+CCR7−; true naïve T cells (TN): CD45RA+CCR7+; central memory T cells (TCM): CD45RA−CCR7+. Regulatory T cells (Tregs) were identified as CD25brightCD127low. Monocyte subsets were classified as follows: total monocytes: CD14+; non-classical monocytes: CD14lowCD16bright; intermediate monocytes: CD14+CD16+; classical monocytes: CD14+CD16−. Dendritic cells (DCs) compartment included: non-dendritic cells (nDCs): CD123−CD11c−; plasmacytoid dendritic cells (pDCs): CD123+CD11c−; myeloid dendritic cells (mDCs): CD11c+CD123−. Details are described in Methods S3 and Tables S3 and S4.

### Data acquisition

Flow cytometry data were acquired on a BD LSR Fortessa SORP cytometer (BD Biosciences, San Jose, CA, USA) using standardized instrument settings and single-color controls for spectral compensation. Daily and weekly quality controls were performed following BD guidelines. Details are described in Methods S4.

### Processing data and statistical analysis

Flow cytometry files were analyzed using FlowJo software v10.8.1 (BD Biosciences, San Jose, CA, USA). Cytokine responses were background-subtracted, and integrated mean fluorescence intensity (iMFI) values were calculated (Methods S5 and Figures S2–S4).

Statistical analyses were conducted using R (R Foundation for Statistical Computing, Vienna, Austria), IBM SPSS Statistics v26 (Chicago, IL, USA), and GraphPad Prism v8.0 (San Diego, CA, USA). Parametric or non-parametric tests were applied as appropriate. Linear models (ANCOVA) were used to assess the independent effects of age, sex, CMV serostatus, and prior SARS-CoV-2 infection on vaccine response. Partial correlation analyses (Pearson’s *r*) and associated *p*-values were calculated, controlling for the same covariates. A significance threshold of *p *< .05 was applied. Details are provided in Methods S5.

## Results

In this study, we evaluated the effect of a fourth Pfizer SARS-CoV-2 booster dose on humoral and cellular immune responses in a healthy population, considering age, sex, prior SARS-CoV-2 infection, and CMV serostatus, and the impact of immunosenescence.

### Humoral response

**Figure 1:**
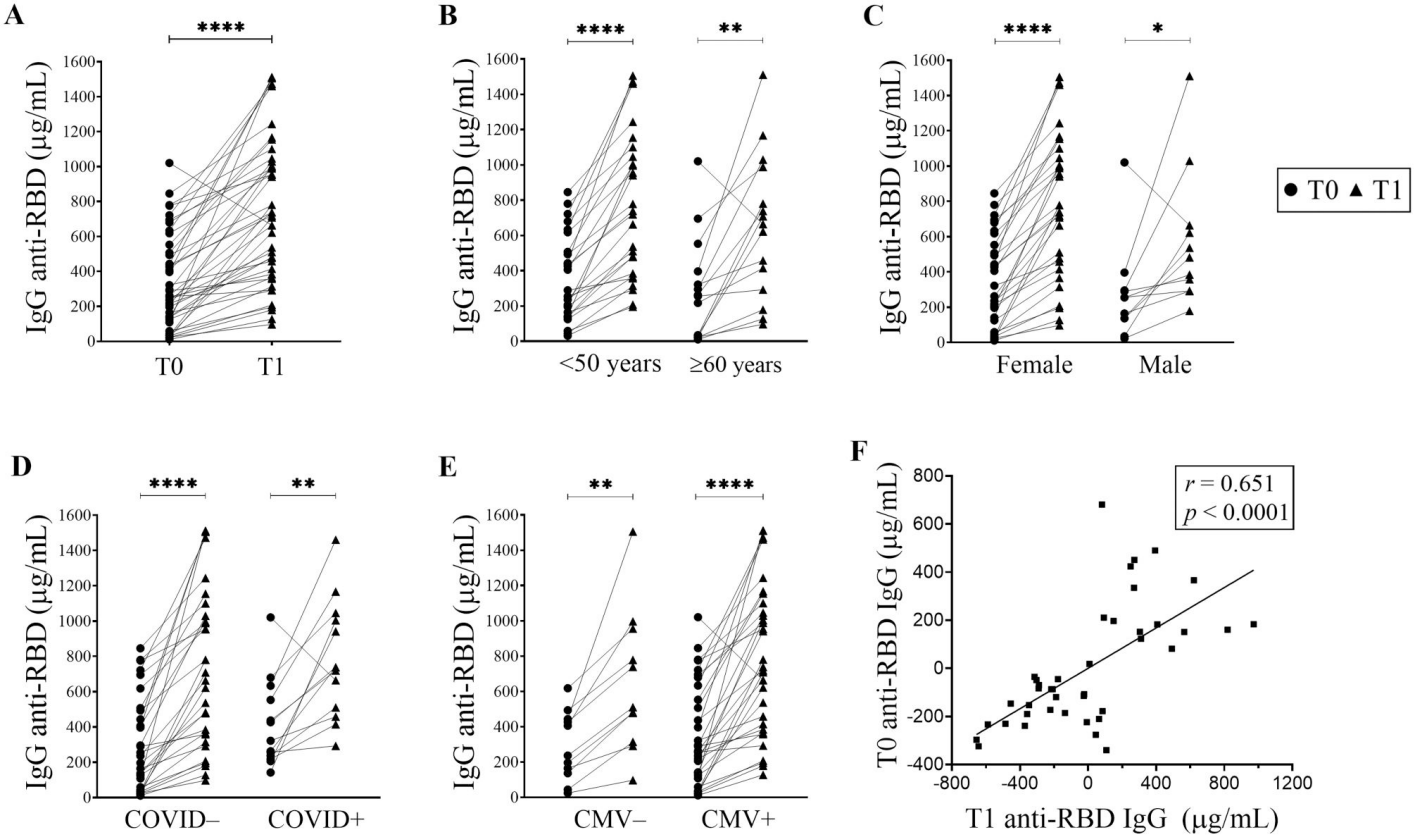
Humoral response to the booster vaccine dose. Responses in the overall cohort (A) and within sub-cohorts defined by age (B), sex (C), prior SARS-CoV-2 infection (D), and CMV serostatus (E). *p < .05; **p < .01; ****p < .0001. (paired *t*-test or Wilcoxon signed-rank test following the Shapiro–Wilk test). (F) Partial correlations (Pearson’s *r*) between SARS-CoV-2 anti-RBD IgG at T0 and T1. Residuals were derived from linear regression models adjusted for age, sex, CMV serostatus, and prior SARS-CoV-2 infection. Degrees of freedom = 41. T0 = pre-boost; T1 = post-boost.

All individuals in our cohort except one (40 out of 41) showed an increase in anti-RBD IgG levels after the booster dose (T1) (Figure 1A). Consistently, when analyzed at the group level, anti-RBD IgG levels increased across all examined sub-cohorts defined by age, sex, prior SARS-CoV-2 infection, or CMV serostatus (Figure 1B–E).

In adjusted linear models, the magnitude of the antibody increase was not significantly affected by any of the tested clinical or demographic covariates (Table S5). Likewise, none of the covariates had a significant effect on antibody levels at T0 and T1, which were also analyzed in the model (Table S5).

Furthermore, partial correlation analysis revealed that baseline antibody levels were positively associated with post-booster titers (Figure 1F). Overall, these results indicate that, in our cohort, booster-induced humoral responses were not significantly driven by age, sex, prior SARS-CoV-2 infection, or CMV serostatus, and that low pre-dose antibody levels were associated with lower post-boost humoral levels.

### Cellular response

**Figure 2:**
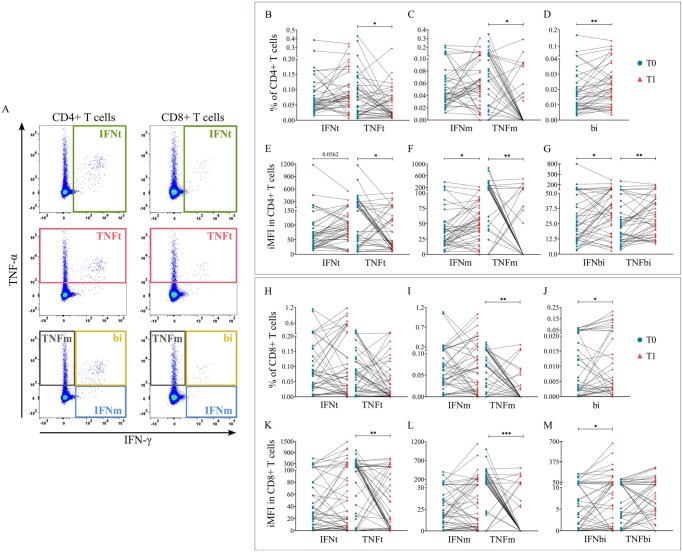
CD4+ and CD8+ T-cell responses to SARS-CoV-2 in the overall cohort. (A) Cellular responses. IFNt: total IFN-γ+ cells (IFN-γ+TNF-α+/−); TNFt: total TNF-α+ cells (TNF-α+IFN-γ+/−); mono-functional cells (IFNm: IFN-γ+TNF-α−; TNFm: TNF-α+IFN-γ−); and bifunctional cells (bi: IFN-γ+TNF-α+). (B–G) CD4+ T-cell responses to SARS-CoV-2 in the overall cohort. (H–M) CD8+ T-cell responses to SARS-CoV-2 in the overall cohort. Circles denote pre-boost (T0) responses and triangles denote post-boost (T1) responses. Lines connect paired observations from the same individual. *p < .05; **p < .01; ***p < .001 (Wilcoxon signed-rank test following the Shapiro–Wilk test).

Cellular response was measured by analyzing IFN-γ and TNF-α production in T cells stimulated with a SARS-CoV-2 spike peptide pool before (T0) and after (T1) the booster dose. Within each T-cell subset (CD4+ and CD8+), we assessed the percentage of total IFN-γ+ cells (IFNt: IFN-γ+TNF-α+/−), total TNF-α+ cells (TNFt: TNF-α+IFN-γ+/−), as well as mono-functional (IFNm: IFN-γ+TNF-α−; TNFm: TNF-α+IFN-γ−) and bi-functional (bi: IFN-γ+TNF-α+) T cells (Figure 2A). Additionally, we analyzed the integrated mean fluorescence intensity (iMFI) of IFN-γ and TNF-α to determine the per-cell cytokine expression. Paired analysis of cytokine levels was performed in the overall cohort, followed by linear models (ANCOVA) to assess the effects of age, sex, prior SARS-CoV-2 infection, and CMV serostatus on these cytokine outputs. Statistical significance of adjusted effects was evaluated using Type III sums of squares (SS) ANOVA (Methods S5). All linear model results are detailed in Table S5 (Excel file).

After the booster, paired analysis in the overall cohort revealed that there was an increased frequency of CD4+bi T cells, along with higher IFN-γ-iMFI in all subsets (Figure 2D–G). Contrarily, the percentages of TNFt and TNFm CD4+ T cells decreased (Figure 2B and C), as well as the TNF-α-iMFI (Figure 2E and F). These findings suggest an increase in IFN-γ-dominant functional responses together with reduced TNF-α–associated inflammatory responses of CD4+ T cells.

Within the CD8+ compartment (Figure 2H–M), after the booster, we found a higher frequency of bi cells accompanied by higher IFN-γ-iMFI (Figure 2J and M). Conversely, decreases were observed in the percentages of TNFt and TNFm cells (Figure 2H and I) and in their TNF-α-iMFI (Figure 2K and L).

**Figure 3:**
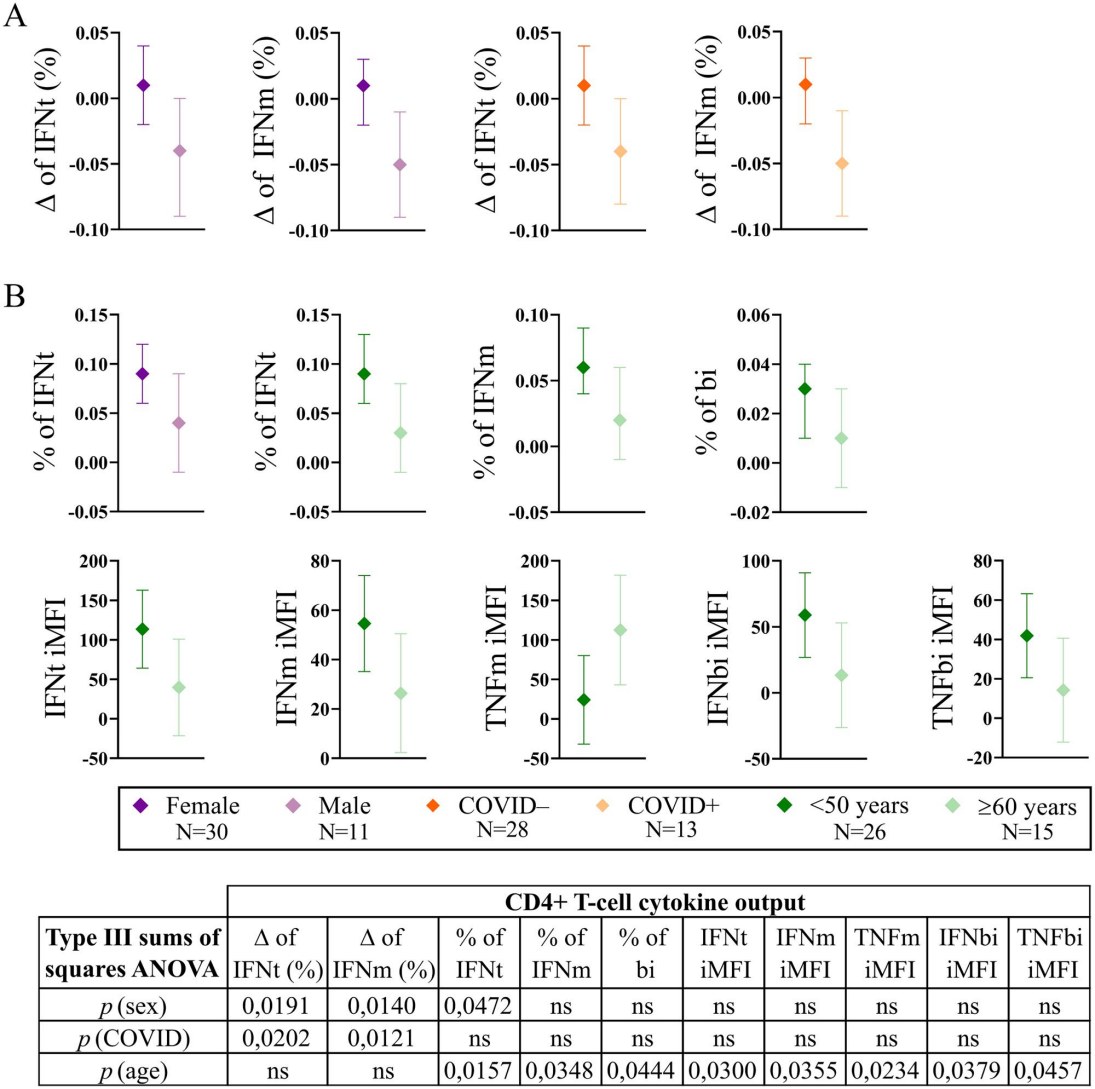
Effect of covariates on CD4+ T-cell cytokine outputs following ANCOVA. (A) Effect of sex and prior SARS-CoV-2 infection (COVID) on changes (Δ) in CD4+ IFNt and IFNm responses. (B) Effect of sex and age on CD4+ T-cell responses after the booster (T1), including cytokine percentages and their iMFI. For each cytokine output, symbols represent estimated marginal means for the sub-cohort groups, with 95% confidence intervals (CIs) shown as horizontal lines. *p*-values in the embedded table indicate the effect of each covariate on cytokine outputs and were obtained from Type III ANOVA (sums of squares). ns = not significant (*p *> .05).

Linear models revealed that, in contrast to the humoral response, the cellular response was shaped by sex and infection history after adjustment for the other covariates. Female sex and absence of prior SARS-CoV-2 infection were independently associated with positive Δ values in the percentage of IFNt and IFNm CD4+ T cells, whereas male sex and prior SARS-CoV-2 infection were associated with negative Δ values (Figure 3A). No effects were observed in Δ values for age or CMV serostatus. TNF-α production was not significantly associated with any of the variables included in the model, indicating that, unlike IFN-γ responses, TNF-α changes were not modulated by these variables. In CD8+ T cells, no significant associations were observed for any of the cytokines analyzed.

To further characterize these associations, we applied analogous ANCOVA models using cytokine percentages at T0 and at T1 as dependent variables. No significant adjusted effects were observed at T0. In contrast, at T1, sex was significantly associated with the percentage of IFNt CD4+ T cells, with male individuals exhibiting lower estimated marginal means (EMMs) compared to females (Figure 3B). Furthermore, linear models showed that increasing age was significantly associated with lower percentages of IFNt, IFNm, and bifunctional CD4+ T cells, and with the corresponding IFN-γ and TNF-α iMFI values, but with higher TNFm iMFI (Figure 3B). No significant effects of CMV serostatus were observed.

These findings suggest that older age, male sex, and prior SARS-CoV-2 infection may be associated with lower CD4+ T-cell responses following vaccination, while CD8+ T-cell responses were not significantly influenced by any of the covariates included in the model.

### Post-booster CD4+IFN-γ+ T-cell phenotypic profile

Since most differences were related to IFN-γ production by CD4+ T cells at T1, we investigated whether their phenotypic profile differed between T0 and T1 in the cohort and sub-cohorts. Co-expression combinations of CD27, CD28, CD56, CD57, and granzyme B (GZB) within IFNt CD4+ T cells were analyzed by SPICE software. The SPICE permutation test evaluates the overall compositional pattern of marker expression (phenotypic profile) rather than each individual slice (phenotypic category) separately. Interpretation is therefore based on shifts in the global distribution of marker combinations across the full pie, which reflect coordinated phenotypic changes. In practice, these differences are more reliably appreciated by examining changes in the arc distributions than by focusing on single slices, which should not be overinterpreted in isolation.

**Figure 4:**
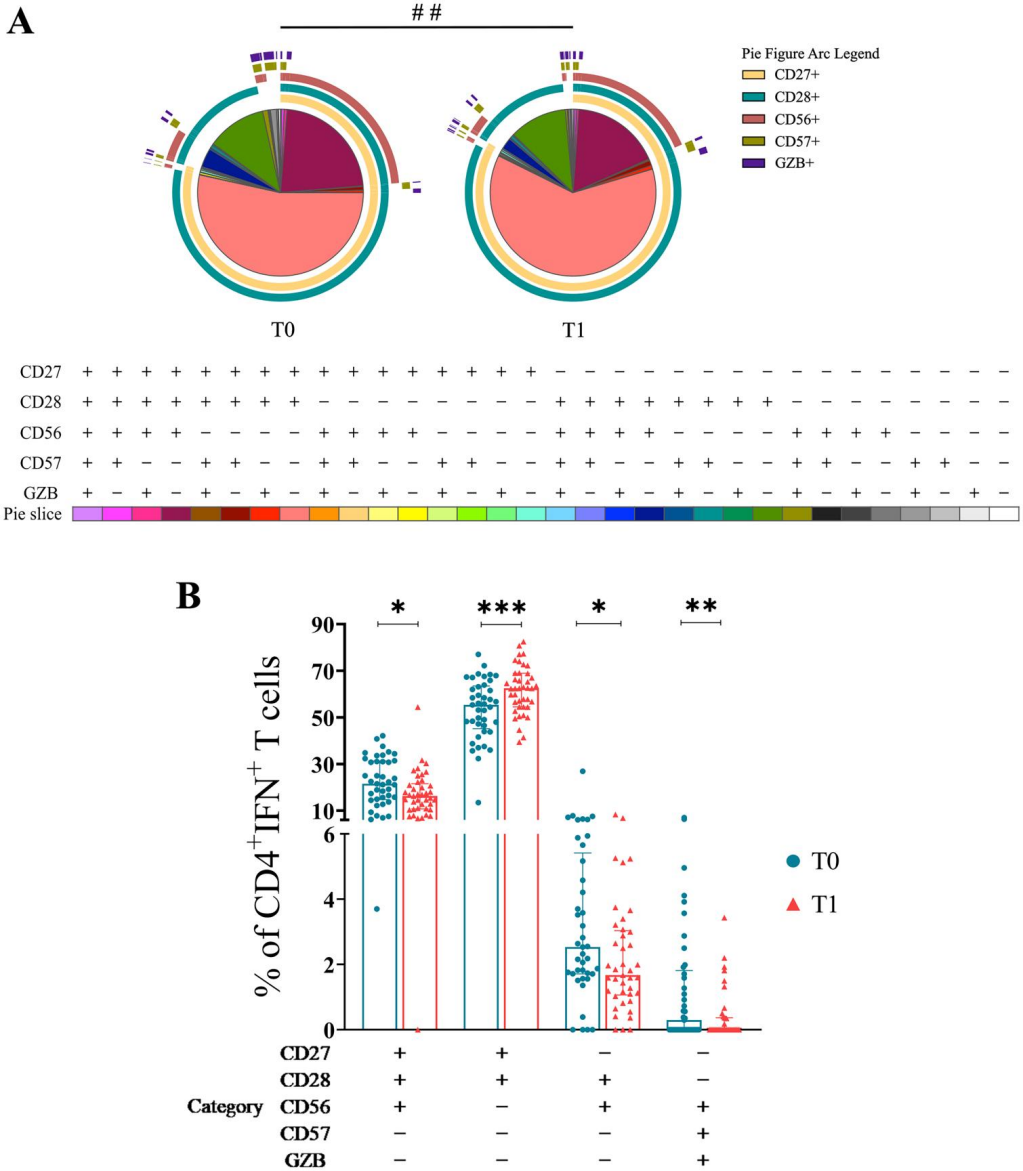
Phenotypic profiles of IFNt CD4+ T cells pre- and post-booster. Pie charts (A) depict the phenotypic profile of CD4+IFN-γ+ T cells in the total cohort at T0 (pie 1) and T1 (pie 2). Each of the 32 slices represents one combination of CD27, CD28, CD56, CD57, and GZB markers, with legends shown below the charts. Marker expression is indicated by a plus sign (+) or a minus sign (−). Pie arcs correspond to individual markers; overlapping arcs indicate co-expression. Significantly different combinations (slices) are shown in the scatter plot (B), where T0 is represented by circles and T1 by triangles. T0 = pre-boost; T1 = post-boost. Bars indicate medians, and whiskers represent the interquartile range (25th–75th percentile). A hash symbol (#) denotes significance by permutation test (^##^*p *< .01), indicating differences in phenotypic profile, while asterisks (*) indicate significance by Wilcoxon signed-rank test (*p < .05; **p < .01; ***p < .001).

Results showed a post-booster phenotype shift toward less differentiation and lower cytotoxicity (Figure 4A). Specifically, there was an increase in the proportion of CD27+CD28+CD56−CD57−GZB− phenotype (*p *< .001), with a concomitant decrease in more differentiated phenotypes (Figure 4B). Sub-cohorts’ analysis revealed a similar pattern in all groups except for male individuals (Figure S5A–D and Table S6), where no post-boost phenotypic change was observed. Notably, this group has a limited sample size. Sub-cohorts’ comparison between groups, at T0 or T1, showed no differences. To improve the interpretability of the SPICE results, heatmaps were generated showing the percentage of each marker combination expressed in each individual before and after the boost, with additional heatmaps produced for the sub-cohort groups (Figure S6).

### Humoral and cellular response correlation to the booster

Vaccine efficacy is generally evaluated based solely on humoral immunity, while the cellular response is overlooked. However, effective antiviral protection requires both robust antibody and T-cell responses. In our cohort, nearly all participants (40 out of 41) showed an increase in anti-RBD IgG levels after the booster, whereas the cellular response was influenced by clinical and demographic conditions. Thus, we performed partial correlation analyses between anti-RBD IgG levels and each T-cell cytokine readout at both T0 and T1, as well as the changes (Δ) in both responses, controlling for sex, age, prior SARS-CoV-2 infection, and CMV serostatus.

This analysis showed significant correlations between pre-booster anti-RBD IgG levels and IFN-γ production in CD4+ T cells (between T0 HR and T0 CR) (Table 1). Furthermore, pre-booster antibody levels correlated with several CD4+ T-cell cytokine readouts after vaccination (between T0 HR and T1 CR). However, only weak correlations were observed between humoral and CD4+ T-cell responses post-booster (between T1 HR and T1 CR), whereas no correlations were found between their respective changes (between ΔHR and ΔCR).

**Table 1:** Partial correlation analysis between humoral response (HR, anti-RBD IgG) and CD4+ T-cell cellular responses (CR).

CD4+ cellular response (CR) readout	Statistics	Humoral response (HR), anti-RBD IgG antibodies			
		ΔHR-ΔCR	T0 HR-T0 CR	T1 HR-T1 CR	T0 HR-T1 CR
% IFNt	Correlation (*r*)	.059	.486**	0	.357*
	*p*-value	.730	.002	.174	.030
% TNFt	Correlation (*r*)	.231	.192	−.056	−.022
	*p*-value	.170	.254	.740	.897
iMFI IFNt	Correlation (*r*)	.115	.387*	.361*	.470**
	*p*-value	.500	.018	.028	.003
iMFI TNFt	Correlation (*r*)	.256	.265	−.093	−.112
	*p*-value	.127	.113	.586	.508
% IFNm	Correlation (*r*)	.004	**.490** **	.118	.238
	*p*-value	.982	.002	.487	.157
% TNFm	Correlation (*r*)	.205	.092	−.237	−.244
	*p*-value	.223	.590	.158	.145
% bi	Correlation (*r*)	.204	.392*	.349*	.437**
	*p*-value	.225	.016	.034	.007
iMFI IFNm	Correlation (*r*)	.071	.397*	.395*	.500**
	*p*-value	.676	.015	.016	.002
iMFI TNFm	Correlation (*r*)	.245	.207	−.233	−.285
	*p*-value	.144	.220	.165	.087
iMFI IFNbi	Correlation (*r*)	.125	.365*	.317	.420**
	*p*-value	.462	.026	.056	.010
iMFI TNFbi	Correlation (*r*)	.200	.390*	.343*	.422**
	*p*-value	.236	.017	.037	.009

Partial correlation coefficients (Pearson’s *r*) and associated *p*-values were calculated between humoral response (HR, anti-RBD IgG antibodies) and CD4+ T-cell responses (CR), at T0, T1, or as increments (Δ) for each CD4+ functional readout listed in the left column of the table. Partial correlations were adjusted for sex, prior SARS-CoV-2 infection, and CMV serostatus. Degrees of freedom = 35.

Abbreviations: Δ = increment; CR = cellular response; HR = humoral response; T0 = before boost; T1 = after boost. *p*-values are two-tailed. Values highlighted in bold denote statistical significance.

* *p *< .05,

** *p *< .01.

Thus, the magnitude of the humoral response post-booster—typically used as a measure of vaccine efficacy—does not necessarily reflect the extent of cellular immune activation. Contrarily, pre-boosting antibody levels may have predictive value for both humoral and cellular responses post-vaccination, regardless of sex, age, CMV serostatus, and prior SARS-CoV-2 infection. Finally, no correlation was observed, pre- or post-booster, between the humoral and CD8+ T-cell responses (Table S7).

### Immunosenescence and vaccination

To investigate if immunosenescence could influence vaccine responses, as previously suggested,^15^ we analyzed senescence-associated markers (Tables S3 and S4) in both innate and adaptive immune cells pre-booster. Immunosenescence level among sub-cohort groups was adjusted by linear models (ANCOVA) evaluating the independent effects of age, sex, prior SARS-CoV-2 infection, and CMV serostatus on immune cell subsets (Tables S5 and S8). Then, we examined whether these immunosenescent phenotypes were associated with the magnitude and quality of the immune response to booster vaccination (Tables S9 and S10).

Linear models showed that older age was associated with a higher percentage of total Tregs (*p *= .0341), CD8+CX3CR1+ T cells, TCM (*p *= .0243), and TEMRA (*p *= .0361) CD8+ T cells (Table S8). Contrarily, younger age was associated with less differentiated phenotypes, including CD4+CD127+ (*p *= .0047), Tregs TN (*p *= .0229), and CD8+ TN (*p *= .004). Surprisingly, we observed an unexpectedly significantly higher CD4:CD8 ratio EMM in individuals over 60 years compared with those under 50. Age also impacted the innate compartment. Specifically, increasing age was associated with higher frequencies of monocytes (*p *= .0105) and NK cells (*p *= .0106), primarily driven by the expansion of the CD56dimCD16+ subset (*p *= .0202). Moreover, the proportion of B cells was influenced by sex (*p *= .0145), with males exhibiting higher adjusted frequencies than females (Table S8).

Demographic statistics state that CMV prevalence increases with age, and it can be reflected in the mean age of CMV− and CMV+ individuals in our cohort (36.2 vs. 52.2 years, Table S1). However, the mean age of CMV+ individuals is markedly lower than that of the older-age group (52.5 vs. 71.5 years, *p *= .006). Despite that, linear models showed that CMV seropositivity associates with several immunosenescence markers, independently of other factors, including age (Table S8). This effect can be observed on proatherogenic CD4+ and CD8+CD28nullCD57+ T cells (*p *= .0208; *p *= .0042), memory-like CD56dim NKG2C+CD57+ NK cells (*p *= .0088), CD4+ T cells expressing the vascular homing receptor CX3CR1 (*p *= .0217), and with a CD4:CD8 ratio inversion (*p *= .0102). These findings are consistent with prior reports identifying CMV as a major contributor to immune aging.^19^

Finally, linear models also showed an association between prior SARS-CoV-2 infection and higher proportions of differentiated subsets, including CD4+ TEMRA cells (*p *= .0003), Treg TEMRA cells (*p *= .000), and CD8+ TCM cells (*p *= .015) (Table S8). In contrast, COVID− individuals showed higher adjusted levels of total Tregs (*p *= .008), CD4+CD25+ cells (*p *= .000), and CD4+ TCM (*p *= .008).

Partial correlation analysis between immunosenescence markers and the humoral response did not show significant associations between antibody titers and any of the senescent phenotypes (Table S9). However, when we analyzed the correlation between the cellular response and the phenotype, we found that the percentage of NKT-like cells (CD8+CD56+), CD8+CX3CR1+, and CD8+CD28^null^CD57+ T cells showed a negative correlation with the CD8+ T-cell response after the booster (Table S10).

### Impact of anti-CMV IgG levels on cellular and humoral immunity to SARS-CoV-2

We next analyzed, within the CMV+ individuals, the partial correlation between anti-CMV IgG antibody levels and both humoral and cellular responses to SARS-CoV-2 peptides, before and after the booster dose, controlling for age, sex, and prior SARS-CoV-2 infection. No significant correlation was observed between anti-CMV IgG titers and the humoral response at either T0 or T1 (Figure S7). Similarly, anti-CMV antibodies did not correlate with the cellular response at T0 (Table S11). However, a significant inverse correlation was found between anti-CMV IgG levels and the IFN-γ production of CD4+ and CD8+ T cells after the booster dose (Table S12). These findings suggest that the response to CMV infection may be associated with differences in cellular responses to other viral antigens, such as SARS-CoV-2, and to vaccination.

## Discussion

Vaccine efficacy evaluation has traditionally relied on the quantification of humoral immunity,^23^ providing only a partial view of the antiviral response, as it neglects the contribution of cellular immunity, which is essential for viral clearance, long-term protection, and cross-reactive defense against emerging variants. Growing evidence underscores that both arms of the adaptive immune system—humoral and cellular—must be considered to comprehensively assess vaccine-induced protection.^24^

In this exploratory study, we addressed this critical gap by simultaneously analyzing humoral and cellular responses to the BNT162b2 mRNA vaccine, before and after administration of a fourth dose (booster). We also accounted for key modifying factors such as age, sex, and latent viral infection, which are determinants of immune responsiveness. Thus, our study provides additional insights into the complexity of vaccine-induced immunity and highlights the need for more holistic approaches in evaluating vaccine effectiveness.

In concordance with previous studies,^6^^,^^17^^,^^20^^,^^25^ we observed a general increment of antibodies against SARS-CoV-2 RBD protein post-booster, independently of age, sex, CMV serostatus, or previous SARS-CoV-2 infection. The increase in antibody levels has been used as the sole indicator of vaccine effectiveness, while their subsequent decline over time has served as the main criterion for recommending additional booster doses.^23^ However, cellular immunity, particularly that elicited after Pfizer mRNA vaccination, persists longer than the humoral response and provides greater protection against variants of concern (VOCs) than neutralizing antibodies.^24^^,^^26^

Our data shows an increase in CD4+ T-cell response (IFN-γ) post-booster, which is a hallmark of antigen-specific antiviral immunity and correlates with viral clearance and vaccine efficacy.^27^ Increases in the percentage of bifunctional CD4+ T cells (IFN-γ+TNF-α+) indicate greater antigen specificity and are associated with more effective and durable protection across different viral infections, including SARS-CoV-2 and influenza.^28^^,^^29^ In contrast, there was a post-booster decrease of CD4+ T-cell TNF-α response. While TNF-α is important in supporting T-cell activation and early inflammatory responses, excessive or sustained TNF-α production has been linked to pathological inflammation and tissue damage during viral infections.^30^^,^^31^ Therefore, the maintenance of a balanced cytokine profile—characterized by the presence of both IFN-γ+ and IFN-γ+TNF-α+ subsets, but without excessive TNF-α dominance—likely reflects an optimal immune state that combines specificity with controlled inflammation.

Notably, the divergent behavior of IFN-γ and TNF-α responses may reflect qualitative functional tuning of recall CD4+ T cells after repeated antigen exposure, favoring antiviral effector functions while limiting pro-inflammatory output. In our study design, the pre-booster measurement was obtained approximately one year after the previous dose, whereas the post-booster assessment was performed 3–5 weeks after the fourth dose. In this context, the observed pattern of TNF-α production is unlikely to be explained solely by missing an early transient TNF-α peak. Nevertheless, differences in cytokine kinetics cannot be completely excluded and therefore represent a limitation of the study.

Post-booster CD8+ T-cell IFN-γ production remained stable, while TNF-α production was decreased. Our data support previous results showing that CD8+ T cells preferentially recognize epitopes from the SARS-CoV-2 nucleocapsid rather than the spike.^26^^,^^29^ Nonetheless, our results show a consistent post-booster decrease in TNF-α production, both total and monofunctional. Given that CD8+ T cells are major cytotoxic effectors^27^ and that TNF-α, while essential for early antiviral defense, can contribute to hyperinflammation and tissue injury when produced in excess,^31^ the observed modulation of TNF-α responses after the booster is consistent with a more regulated inflammatory profile. Our findings suggest that booster vaccination may be associated with a shift in CD8+ T-cell functional profile, favoring a more balanced and potentially protective immune profile.

However, despite this general pattern of T-cell responses, we observed that the cellular cytokine production was influenced by sex and SARS-CoV-2 infection history. Specifically, male participants and previously infected individuals showed a post-booster decrease in IFN-γ–producing CD4+ T cells, which is consistent with previous studies.^6^^,^^32^^,^^33^

Only a few studies have specifically addressed the possibility of immune exhaustion in the context of recurrent booster vaccination. No references about this aspect have been found in individuals with previous SARS-CoV-2 infection who have received 4 doses of BNT162b2. Some studies did not find signs of exhaustion after booster doses; their cohorts included only SARS-CoV-2 naïve participants and/or heterologous administration of both Pfizer and Moderna mRNA vaccines.^12^^,^^34^ Although exhaustion markers were not analyzed in this work, our results suggest that breakthrough infections followed by 2 booster doses of BNT162b2 were associated with lower specific CD4+ T-cell responses. Previous analysis of the third dose concluded that the heterologous vaccination regimen achieves greater responses than homologous schedules.^35^ In Spain, Pfizer BNT162b2 has been the most administered vaccine (74.2%), especially among healthcare workers and older adults.^36^ Taken together, the results obtained in the present study may be relevant for a substantial proportion of similarly vaccinated populations. This wane of specific CD4+ T-cell response highlights the importance of the recent research regarding the development of new adjuvants that enhance not only the humoral but also the cellular immunity.^37^

We further examined whether the CD4+IFN-γ+ T cells changed their phenotypic profile in response to vaccination. This analysis showed that post-booster CD4+ T-cell responses shifted toward a less differentiated and less cytotoxic profile. However, this shift was absent in male individuals. A shift toward a less differentiated and less cytotoxic phenotype among CD4+IFN-γ+ T-cell responders after the booster is consistent with a less differentiated profile, typically associated with greater proliferative potential and longevity that are linked to durable memory after vaccination.^21^ Conversely, accumulation of late differentiated and cytotoxic CD4+ populations—often marked by CD27/CD28 loss and CD57 expression—has been associated with impaired recall and proliferative capacity.^21^ Aging studies further show a bias toward more differentiated T-cell states with reduced functional reserve, underscoring the disadvantage of terminal phenotypes.^38^ Thus, failure to exhibit this shift—as observed in males—could contribute to their comparatively weaker CD4+ responses.

When exploring the relationship between humoral and cellular immunity, we found that individuals with lower antibody titers pre-booster also exhibited weaker humoral and cellular responses post-booster. Additionally, higher baseline antibody levels correlated with stronger CD4+ T cells post-booster response, suggesting that pre-existing immune memory conditions the magnitude of both arms of the adaptive response upon re-exposure. This pattern indicates a coordinated recall capacity, whereby individuals with more robust baseline immunity are better equipped to mount efficient secondary responses.^39^ Humoral and cellular adaptive responses follow distinct activation kinetics, effector functions, and memory trajectories. Once immune memory is established, antibody and T-cell responses can be regulated partly independently, reflecting their different developmental pathways and maintenance mechanisms.^23^^,^^40–42^ Together, these observations reinforce the importance of assessing both humoral and cellular immunity to gain a comprehensive understanding of vaccine-induced protection.

Importantly, our data highlight the impact of aging and chronic viral exposures on immune homeostasis within our cohort. The accumulation of CD8+ TCM and TEMRA cells, Tregs, and a decline in naïve populations are consistent with established hallmarks of immunosenescence^15^^,^^38^ and suggest a shift toward more differentiated immune profiles and reduced immune plasticity. CMV seropositivity was associated with expansion of CD28nullCD57+ T cells and memory-like NK subsets, phenotypes that have been widely linked to CMV-driven immune remodeling.^21^ Notably, the expansion of CD28nullCD57+ T cells—classically associated with terminal differentiation^43^—was observed primarily in CMV-seropositive individuals rather than as a direct function of chronological age. This pattern was particularly remarkable in the CD4+ compartment, where the CD28nullCD57+ phenotype is typically rare, and is consistent with previous reports^44^^,^^45^ describing CMV-associated differentiation signatures.

Surprisingly, older participants exhibited a higher CD4:CD8 ratio compared with younger individuals. The most plausible explanation would be a relatively low prevalence of CMV infection among older participants, which would limit the CMV-driven expansion of terminally differentiated CD8+ T cells. However, this was not the case in our cohort, in which only one older individual was CMV−. Such a pattern has also been described in healthy aging populations and may reflect preserved immune homeostasis.^19^^,^^38^ In contrast, CMV+ individuals in our cohort did display the characteristic CD4:CD8 inversion, consistent with established features of advanced immunosenescence,^46^^,^^47^ again supporting the role of CMV infection in shaping senescent immune profiles. Furthermore, prior SARS-CoV-2 infection was also linked to phenotypic changes reminiscent of senescence, pointing to the potential long-term imprint of acute viral challenges on immune composition.^48^

When these immunosenescence-associated features were explored in relation to booster vaccine responses, no associations were found with antibody titers, indicating that humoral responses remain relatively preserved. However, the frequency of CD8+CX3CR1+ T cells was associated with lower CD8+ T-cell post-booster response. Interestingly, this subset was significantly influenced by age, with older individuals exhibiting higher percentages than the younger ones. Furthermore, the frequencies of NKT-like (CD8+CD56+) and CD8+CD28nullCD57+ T cells—both hallmarks of CMV-driven differentiation^44^^,^^49^—also correlated negatively with CD8+ T-cell functional post-booster responses. Thus, age and CMV-related immunosenescence may be associated with reduced cellular responses, even in the absence of major alterations in antibody generation. Furthermore, we observed that higher anti-CMV IgG titers were inversely correlated with IFN-γ post-booster production by CD4+ and CD8+ T cells, without association with the humoral response. This association suggests that the intensity of CMV-specific immune activity may be linked to differences in cellular responses to SARS-CoV-2 vaccination. The absence of correlation with antibody titers indicates that this relationship appears to be preferentially restricted to the cellular arm of the immune system and may be consistent with CMV-associated immune remodeling or functional constraints within the T-cell pool. Taken together, our results are compatible with prior reports suggesting that persistent CMV infection may modulate immune function and contribute to inter-individual variability in responses to heterologous antigens, including SARS-CoV-2 vaccination.^19^^,^^21^

Prior studies on CMV and COVID-19 vaccination have focused on serostatus rather than anti-CMV IgG titers, yielding mixed results for cellular and humoral readouts.^18^^,^^20^^,^^50^ To the best of our knowledge, none directly assessed quantitative anti-CMV IgG levels against SARS-CoV-2-specific T-cell IFN-γ after boosting,^51^^,^^52^ underscoring the novelty of our observation.

Despite its strengths, our study has several limitations. The relatively small sample size may limit the statistical power to detect subtle subgroup effects, and our observations should therefore be interpreted with caution. Due to this limitation, our statistical approach focused on detecting the main effects of age, sex, CMV serostatus, and SARS-CoV-2 prior infection rather than on interaction analyses or pairwise comparisons. Larger studies will be needed to formally evaluate these multifactorial interactions. Also, because this is an observational immunophenotyping study, associations should not be interpreted as demonstrating causality, and mechanistic interpretations remain inferential. Moreover, the vaccine formulation evaluated here contained the ancestral SARS-CoV-2 spike antigen, whereas the most recent booster regimens have replaced this component with variant-specific sequences. Although our findings do not indicate booster-induced features of T-cell senescence, repeated stimulation with the same ancestral antigen could, in theory, favor excessive differentiation over time. In this regard, the current strategy of updating booster formulations with circulating variants may help sustain a more functional and diverse T-cell repertoire. Our results reinforce the relevance of maintaining cellular reactivity through timely vaccine updates to ensure broad and durable protection against evolving strains.

A further strength of our study lies in the simultaneous assessment of humoral and cellular immunity, showing that these 2 arms of the adaptive response are not necessarily correlated. This highlights the need to include cellular parameters when evaluating vaccine effectiveness. Additionally, by integrating host factors such as age, biological sex, CMV serostatus, and previous SARS-CoV-2 infection, our findings emphasize that these variables significantly shape both the magnitude and quality of vaccine-induced immunity and should be accounted for.

## Supplementary Material

glag095_Supplementary_Data

## Data Availability

Flow cytometry row data will be made available upon reasonable request.
