# Supplementary material for: Immunosenescence and cytomegalovirus-associated immune signatures on severe acute respiratory syndrome coronavirus 2 booster responses
Source: J Gerontol A Biol Sci Med Sci. 2026 Apr 17;81(5):glag095. doi: 10.1093/gerona/glag095 (PMC13134772; doi:10.1093/gerona/glag095)
Supplement: glag095_Supplementary_Data [file glag095_supplementary_data.zip › CLEAN_SUPP_ReinaI_Gerontology_2025_Rev.pdf]

SUPPLEMENTARY METHODS

Methods 1. SARS-CoV-2 IgG anti-RBD ELISA and anti-CMV IgG determination.

Methods 2. T-cell Functionality Assay.

2.1. PBMC Isolation and Culture.

2.2. Stimulation.

2.3. Surface and Intracellular Staining.

Methods 3. Immune Cell Immunophenotyping.

Methods 4. Data Acquisition.

Methods 5. Processing Data and Statistical Analysis.

SUPPLEMENTARY FIGURES

Figure S1. Participant recruitment flowchart.

Figure S2. Gating strategy for intracellular staining (ICS) panel.

Figure S3. Gating strategy for T cells panel.

Figure S4. Gating strategy for innate immune cells panel.

Figure S5. CD4+IFN-γ+ T-cell phenotype before (T0) and after (T1) the booster dose across sub-cohorts.

Figure S6. Heatmap representation of Boolean-derived phenotypic marker combinations for CD4+IFN-γ+ T cells.

Figure S7. Partial correlations between anti-CMV IgG and anti-RBD IgG levels among CMV+ individuals.

SUPPLEMENTARY TABLES

Table S1. Participant demographics.

Table S2. Cytometry panel for functional assays.

Table S3. Cytometry panel for T-cell phenotyping.

Table S4. Cytometry panel for Innate cell phenotyping.

Table S5 Linear models assessing the effects of clinical and demographic covariates on immune phenotypes, antibodies and cytokine changes. (Excel file).

Table S6. Sub-cohort comparisons of CD4+ IFN-γ+ T-cell phenotype between T0 and T1.

Table S7. Partial correlations between humoral and CD8+ T-cell responses.

Table S8. Linear models for clinical and demographic covariates effects on immune phenotypes.

Table S9. Partial correlations between immune phenotypes and anti-RBD IgG titres.

Table S10. Partial correlations between CD8+ T-cell phenotype and CD8+ T-cell response at T1.

Table S11. Partial correlations between anti-CMV IgG and T-cell responses at T0.

Table S12. Partial correlations between anti-CMV IgG and T-cell responses at T1.

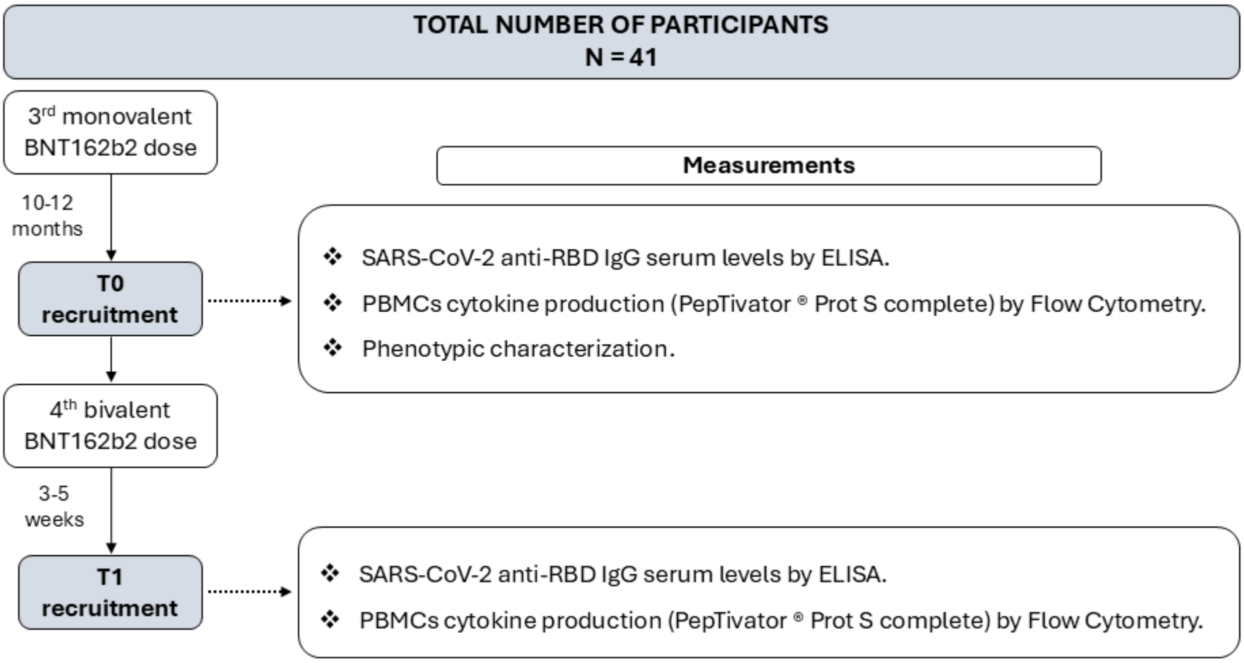

Figure S1. Participant recruitment flowchart. A total of 41 healthy donors were recruited. Sample collection prior to booster administration was performed 10–12 months after the third dose of the monovalent BNT162b2 vaccine (time point 0 [T0]). Following T0 sample collection, participants received a fourth dose of the bivalent BNT162b2 vaccine, and additional samples were collected 3–5 weeks after administration (time point 1 [T1]). Measurements included serum levels of SARS-CoV-2 anti-RBD IgG and cytokine production assays (ICS) at T0 and T1, as well as immunophenotypic characterization of participants at T0 by flow cytometry.

Table S1. Participant demographics.

Sub-cohort	Sub-cohort group	N	Age range (age±SD)	Age group (<50/≥60)	Sex (female/male)	SARS-CoV-2 infection (no/yes)	CMV (−/+)
Total	n/a	41	23-79 (48.1±19.7)	26/15	30/11	28/13	11/30
Age	<50 years	26	23-48 (34.7±9.4)	n/a	21/5	19/7	10/16
	≥60 years	15	60-79 (71.5±5.3)	n/a	9/6	9/6	1/14
Sex	Female	30	23-77 (44.7±19.5)	21/9	n/a	19/11	9/21
	Male	11	31-79 (57.6±17.7)	5/6	n/a	9/2	2/9
SARS-CoV-2 infection	COVID−	28	23-79 (46.8±18.8)	19/9	19/9	n/a	9/19
	COVID+	13	25-77 (51.2±22)	7/6	11/2	n/a	2/11
CMV serostatus	CMV−	11	23-60 (36.2±12.2)	10/1	9/2	9/2	n/a
	CMV+	30	24-79 (52.5±20.3)	16/14	21/9	19/11	n/a

Note. n/a = not applicable.

Methods 1. SARS-CoV-2 IgG anti-RBD ELISA and anti-CMV IgG determination.

For anti–SARS-CoV-2 RBD IgG quantification, 96-well ELISA plates (Nunc Maxisorp, Thermo Fisher Scientific, Cat#442404) were coated overnight at 4 °C with 100 µL of recombinant RBD protein (AcroBiosystems, Cat#SPD-C52H3-100ug) at 1 µg/mL in PBS (PAN-Biotech, Cat#P04-36500). After washing, plates were blocked with 1% BSA in PBST for 1 h at room temperature (RT). Serum samples were serially diluted in PBST + 1% BSA and added in duplicate. A standard curve (Novus Biologicals, Cat#NBP3-05728; 50–0.78 ng/mL) was included in each plate. Samples and standard were incubated for 1 h at 37°C. Following washes, HRP-conjugated anti-human IgG (Invitrogen, Cat#A18903; 1:4000) was added for 1.5 h at RT. Plates were developed with TMB substrate (Hello Bio, Cat#HB8566) for 2.5 min, the reaction stopped with 2N H_2SO_4, and optical density measured at 450/630 nm (Delta Lab plate reader, Cat#MB-580). Concentrations were calculated using each plate's standard curve and corrected by the corresponding dilution factor. Intra- and inter-assay coefficients of variation were 8.3% and 4.2%, respectively.

Anti-CMV IgM and IgG antibodies were determined using chemiluminescent immunoassays (Liaison CMV IgM II and CMV IgG II) on the Liaison XL® system (DiaSorin), performed at the Microbiology Service of Reina Sofía University Hospital (Córdoba, Spain).

Methods 2. T-cell Functionality Assay.

2.1. PBMC Isolation and Culture.

Peripheral blood was collected in lithium heparin tubes and PBMCs were isolated by density-gradient centrifugation using Lymphoprep (Cosmo Bio USA). Ten million fresh PBMCs were kept overnight at 4 °C in TexMACS medium (Miltenyi Biotec) supplemented with 100 U/mL penicillin G and 100 mg/mL streptomycin. The following day, cells were rested for 30 min at 37 °C and viability confirmed. PBMCs (2×10^6 per well) were plated in 96-well U-bottom plates in 200 µL.

2.2. Stimulation.

Cells were stimulated with PepTivator® SARS-CoV-2 S complete peptide pool (Miltenyi Biotec, Cat#130-127-951) according to manufacturer instructions at a final concentration of 0.6 nmol/peptide/mL. Positive (CytoStim, 20 µL/mL) and negative (DMSO 10%, 10 µL/mL) controls were included. After 2 h at 37 °C, 1 µg/mL brefeldin A (BD GolgiPlug, Cat#555029) was added and cells incubated 4 h longer. Cells were washed with cold PBS + 1% FBS and treated with 2.85 mM EDTA for 10 min at 4 °C prior to further washes.

2.3. Surface and Intracellular Staining.

Cells were stained for 30 min at 4 °C with LIVE/DEAD™ Fixable Near-IR dye (Thermo Fisher Scientific) and surface antibodies (**Table S2**). After fixation and permeabilization with Cytofix/Cytoperm™ (BD Biosciences), intracellular cytokine staining (ICS) was performed for 30 min at 4 °C using antibodies listed in **Table S2**. Intracellular anti-CD3 was included to counteract activation-induced downregulation. Cells were finally resuspended in Running Buffer (Miltenyi Biotec) and acquired the following day.

Table S2. Cytometry panel for functional assays.

Antibody	Clon	Fluorochrome	Supplier
**Surface staining**			
Live/Dead Fixable Near-IR Dead Cell Stain Kit	N/A	Near-IR	Molecular Probes of Life Technologies
CD8	RPA-T8	V-500	BD Becton Dickinson
CD4	RPA-T4	BUV395	BD Becton Dickinson
CD57	TB03	APC	BD Becton Dickinson
CD28	CD28.2	PE-Cy7	BD Becton Dickinson
CD27	M-T271	BV711	BD Becton Dickinson
CD45	HI30	Alexa Fluor 700	BD Becton Dickinson
CD56	NCAM16.2	BV421	BD Becton Dickinson
**Intracellular staining (ICS)**			
**Antibody**	**Clon**	**Fluorochrome**	**Supplier**
CD3	SK7	PE-Cy5.5	Invitrogen
IFN-γ	45-15	FITC	Miltenyi Biotec
TNF-α	MAb11	BV650	BD Becton Dickinson
GZB	GB11	PE	BD Becton Dickinson

Methods 3. Immune Cell Immunophenotyping.

Fresh whole blood (100 μL) was stained with monoclonal antibody panels targeting T-cell (**Table S3**) and innate cell subsets (**Table S4**). Samples were incubated for 20 min at room temperature in the dark, followed by red-cell lysis using FACS Lysing Buffer (BD Biosciences). After washing with PBS + 1% FBS, cells were resuspended in 300 μL Running Buffer (Miltenyi Biotec) and acquired within 1–4 h.

Table S3. Cytometry panel for T-cell phenotyping.

Antibody	Clon	Fluorochrome	Supplier
CD45	HI30	Alexa Fluor 700	BD Becton Dickinson
CD3	SK7	PE-Cy5.5	Invitrogen
CD8	SK1	APC-H7	BD Becton Dickinson
CD4	SK3	BB700	BD Becton Dickinson
TCRγδ	B1	BV650	BD Becton Dickinson
CD57	NK-1	FITC	BD Becton Dickinson
CD28	CD28.2	PE-Cy7	BD Becton Dickinson
CX3CR1	2A9-1	PE-CF594	BD Becton Dickinson
CD27	M-T271	APC	BD Becton Dickinson
CCR7	3D12	BV786	BD Becton Dickinson
CD45RA	HI100	BV605	BD Becton Dickinson
CD25	M-A251	PE	BD Becton Dickinson
HLA-DR	L243	BV570	BioLegend
CD127	HIL-7R-M21	BB711	BD Becton Dickinson
CD56	NCAM16.2	BV421	BD Becton Dickinson

Table S4. Cytometry panel for innate cell phenotyping.

Antibody	Clon	Fluorochrome	Supplier
CD57	HNK-1	FITC	BD Becton Dickinson
CD7	M-T701	APC	BD Becton Dickinson
HLA-DR	L243	BV570	BioLegend
CD16	3G8	BV786	BD Becton Dickinson
CD11c	3.9	PE-CF594	BD Becton Dickinson
NKG2C	REA205	PE	Miltenyi Biotec
CD56	NCAM16.2	BV421	BD Becton Dickinson
CD45	HI30	Alexa Fluor 700	BD Becton Dickinson
CD123	7G3	PE-Cy7	BD Becton Dickinson
CD14	TÜK4	APCVio770	Miltenyi Biotec
CD19	SJ25C1	BV650	BD Becton Dickinson
CD3	SK7	PE-Cy5.5	Invitrogen

Methods 4. Data Acquisition.

All samples were acquired using a BD LSR Fortessa SORP flow cytometer. Automatic spectral overlap compensation was performed in BD FACSDiva v8.0.1 (BD Biosciences) using single-color compensation controls. Cytometer performance was monitored weekly with BD CS&T calibration beads, and 8-peak Rainbow Compensation Particles (BD) were run prior to each experiment to ensure stable photomultiplier tube (PMT) settings. PMT voltages were adjusted when necessary to maintain consistent fluorescence detection across experiments.

Methods 5. Processing Data and Statistical Analysis.

Data were analysed using FlowJo v10.8.1 (TreeStar). Gating strategies for functional assays, T-cell phenotyping and innate cell panels are shown in **Figures S2–S4**. For the cytokine analysis (ICS panel), background subtraction was applied using the paired negative control. Integrated Mean Fluorescence Intensity (iMFI) was calculated by multiplying the frequency of the cytokine by its MFI obtained from FlowJo as Geometric Mean. Boolean tool in FlowJo was used to generate combination of markers, including all combinations of CD27, CD28, CD56, CD57 and GZB expression within IFN-γ+ CD4+ T cells. These phenotypic distributions were exported into SPICE v5.35 (NIH) (Roederer M, Nozzi JL, Nason MC. SPICE: exploration and analysis of post-cytometric complex multivariate datasets. Cytometry A. 2011;79(2):167-74), and group comparisons were performed using SPICE permutation tests (10,000 permutations). Significance thresholds were set at #$p < 0.05$ and ##$p < 0.01$ for permutation results. Slice-by-slice comparisons were performed using Wilcoxon signed-rank tests. Asterisks (*) indicate statistical significance as follows: *$p < 0.05$, **$p < 0.01$, ***$p < 0.001$.

Redundancy analysis was applied to T-cell and innate-cell phenotyping datasets to evaluate collinearity and reduce variable redundancy prior to hypothesis testing. Statistical analyses were performed using GraphPad Prism v8.0, IBM SPSS Statistics v26 and R v4.3.1 (packages: tidyverse, car, broom).

For paired samples, normality was assessed using the Shapiro–Wilk test in GraphPad Prism or SPSS. Depending on distribution, comparisons were performed using paired *t*-tests or Wilcoxon signed-rank tests.

Given the limited sample size and the unbalanced distribution of covariates across sub-cohort groups, which precluded reliable pairwise comparisons, we applied analysis of covariance (ANCOVA) within the general linear modelling framework. For each outcome variable, a linear model was fitted including age, sex, prior SARS-CoV-2 infection, and CMV serostatus simultaneously as independent variables, allowing estimation of the independent effect of each factor while adjusting for the remaining covariates. Statistical significance of adjusted effects was assessed using Type III ANOVA (sums of squares), with a significance threshold of $p < 0.05$. Estimated marginal means (EMMs) with a 95% confidence interval (95% CI) were calculated, and the results are presented in **Table S5** (Excel file). Negative EMMs arise from the statistical model fitting process. Except for changes (Δ), which may be negative, EMM values below 0 should be interpreted as very low or near-zero expression, as they do not represent biologically plausible values.

Partial correlations (Pearson's r) and associated p-values were calculated using SPSS controlling for age, sex, prior SARS-CoV-2 infection, and CMV serostatus. For visualization, residuals of each variable were obtained from linear regression models adjusted for the same covariates. p-values are two-tailed.

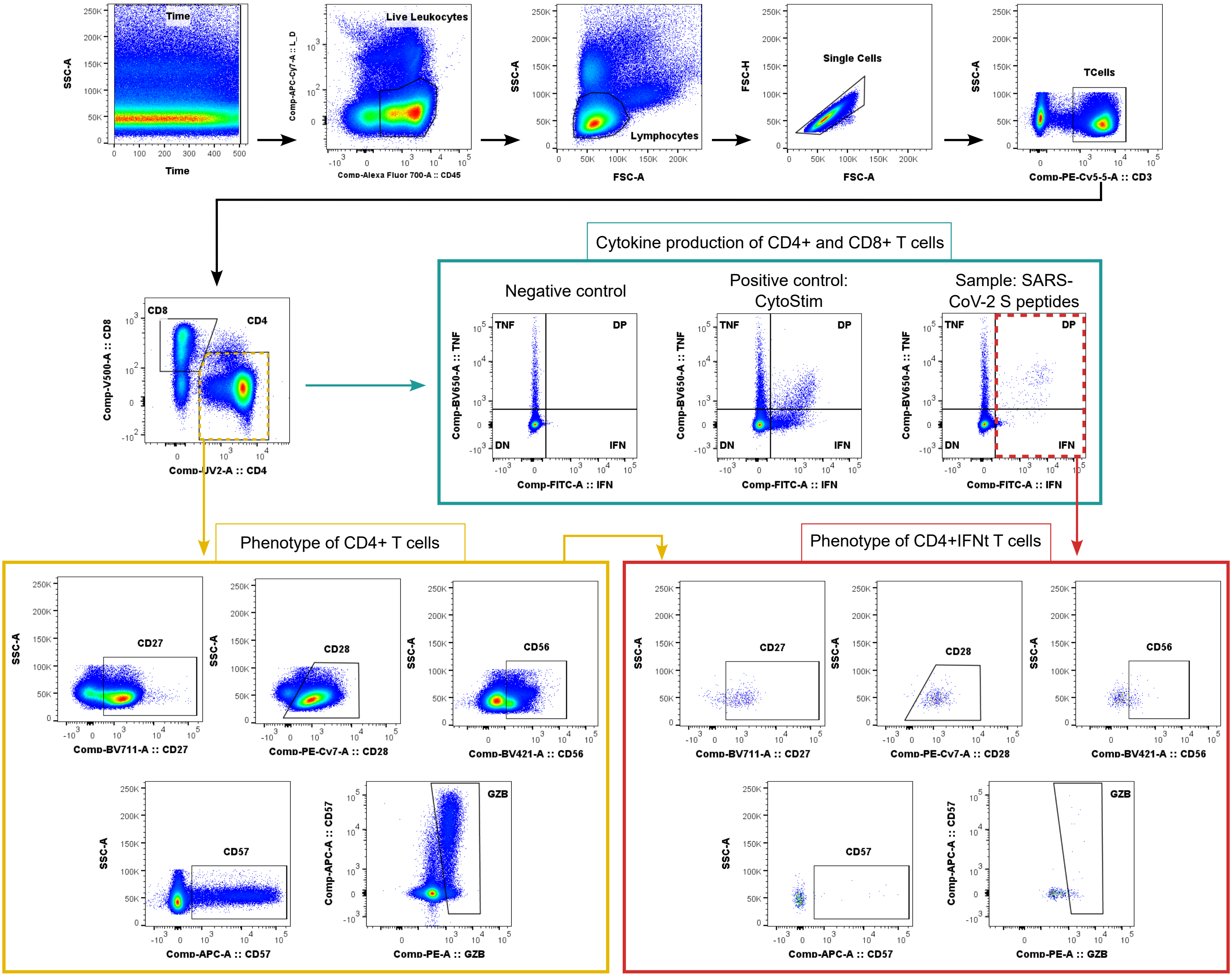

Figure S2. Gating strategy for intracellular staining (ICS) panel. SARS-CoV-2–specific T-cell responses were measured following stimulation with complete spike (S) protein peptide pool. Background subtraction was applied using the paired negative control. The same gates defined for the phenotype of overall CD4+ T cells were applied to the CD4+IFNt population of the peptide stimulated samples. DP: Double Positive cells; DN: Double Negative cells; GZB: Granzyme-B.

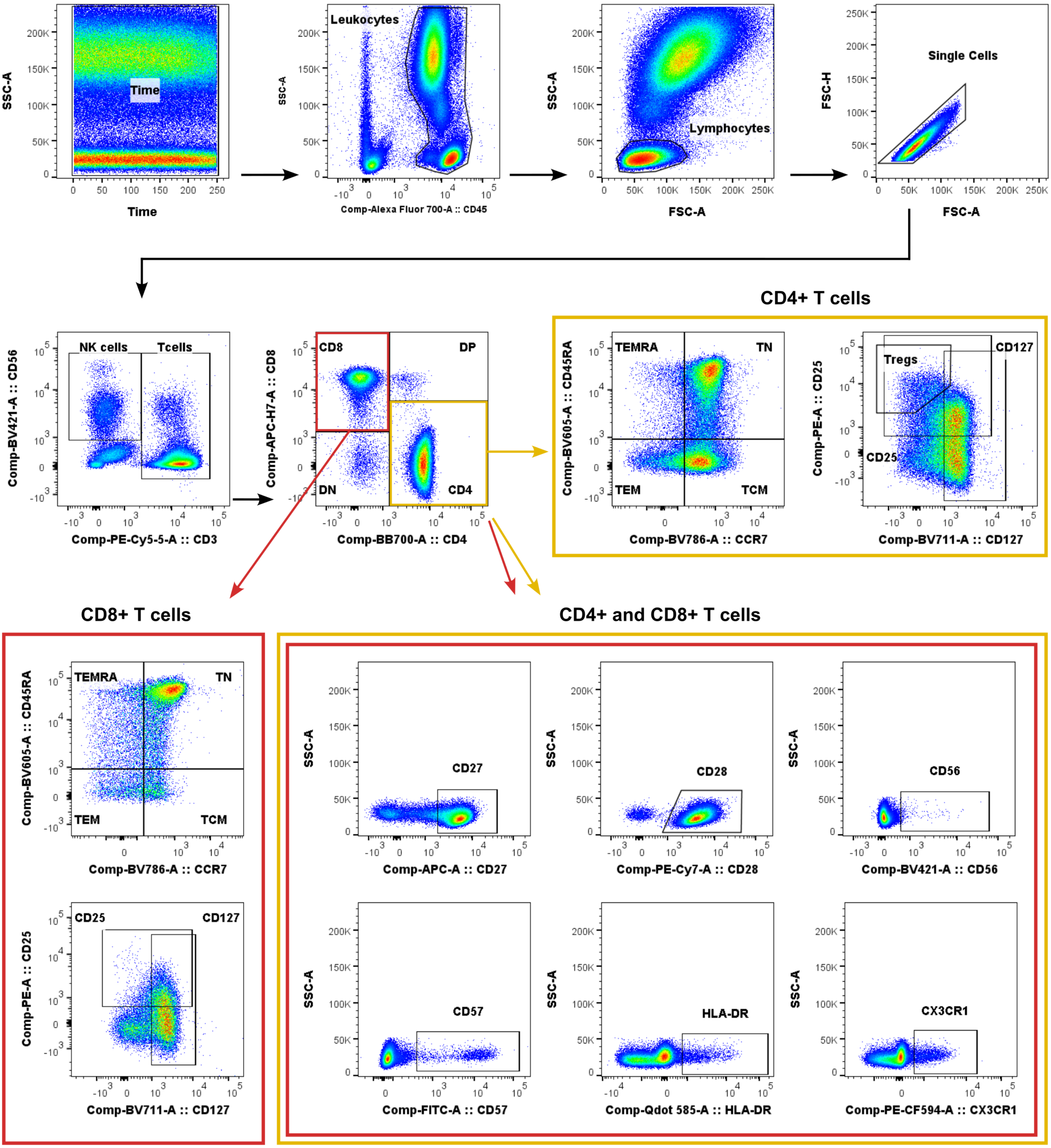

Figure S3. Gating strategy for T cells panel. FlowJo Boolean tool was used to create coexpression patterns for CD27, CD28, CD56, CD57, HLA-DR and CX3CR1 markers. DP: Double Positive cells; DN: Double Negative cells; TEMRA: Terminally Differentiated Effector Memory T cells re-expressing CD45RA; TN: True Naïve T cells; TEM: Effector Memory T cells; TCM: Central Memory T cells; Tregs: Regulatory T cells.

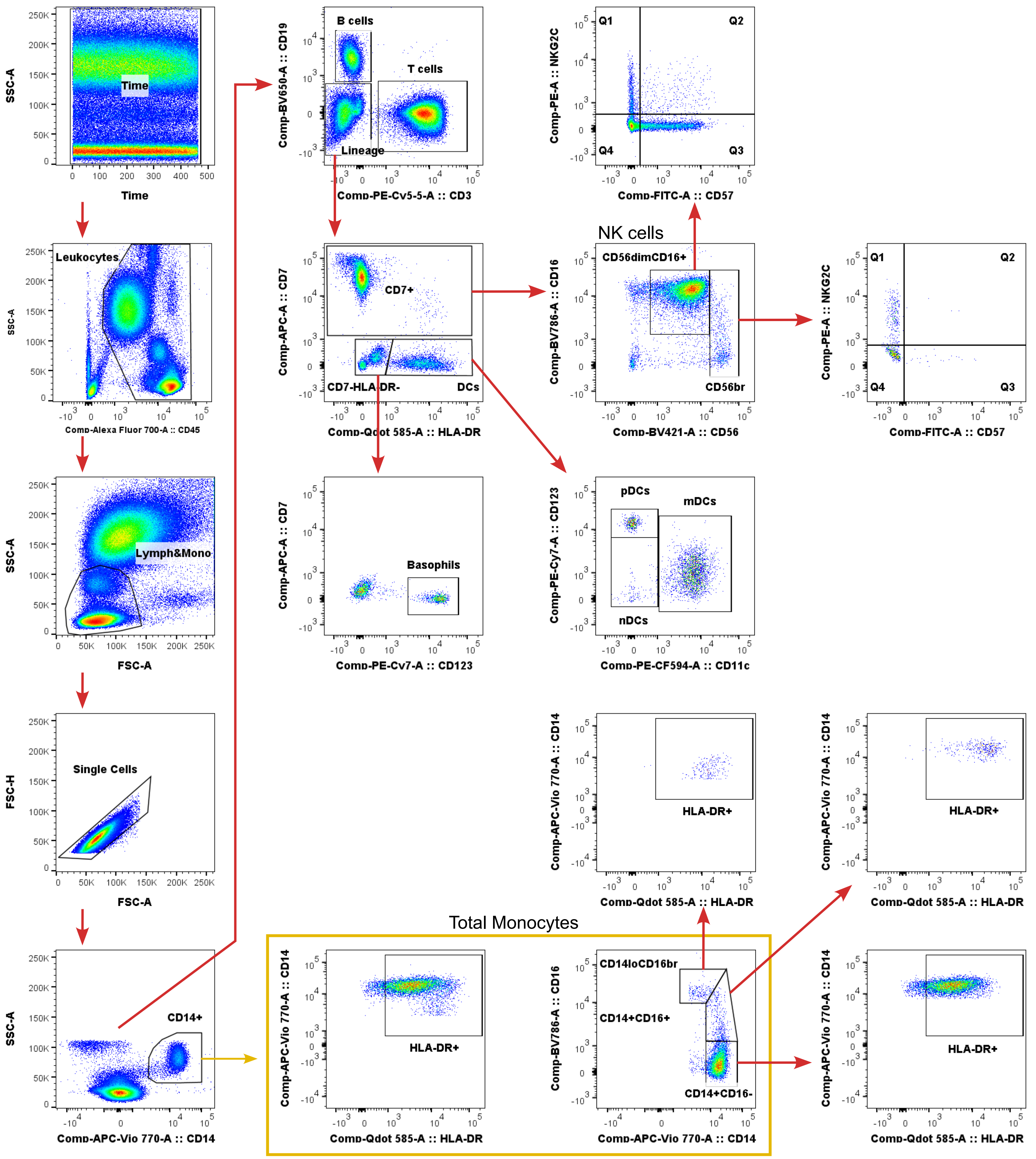

Figure S4. Gating strategy for innate immune cells panel. CD14+: total monocytes; CD14loCD16br: non classical monocytes; CD14+CD16+: intermediate monocytes; CD14+CD16-: classical monocytes; Lineage: lineage cells; CD7+: NK cells; DCs: Dendritic Cells; pDCs: plasmacytoid DCs; mDCs: myeloid DCs; nDCs: double negative DCs.

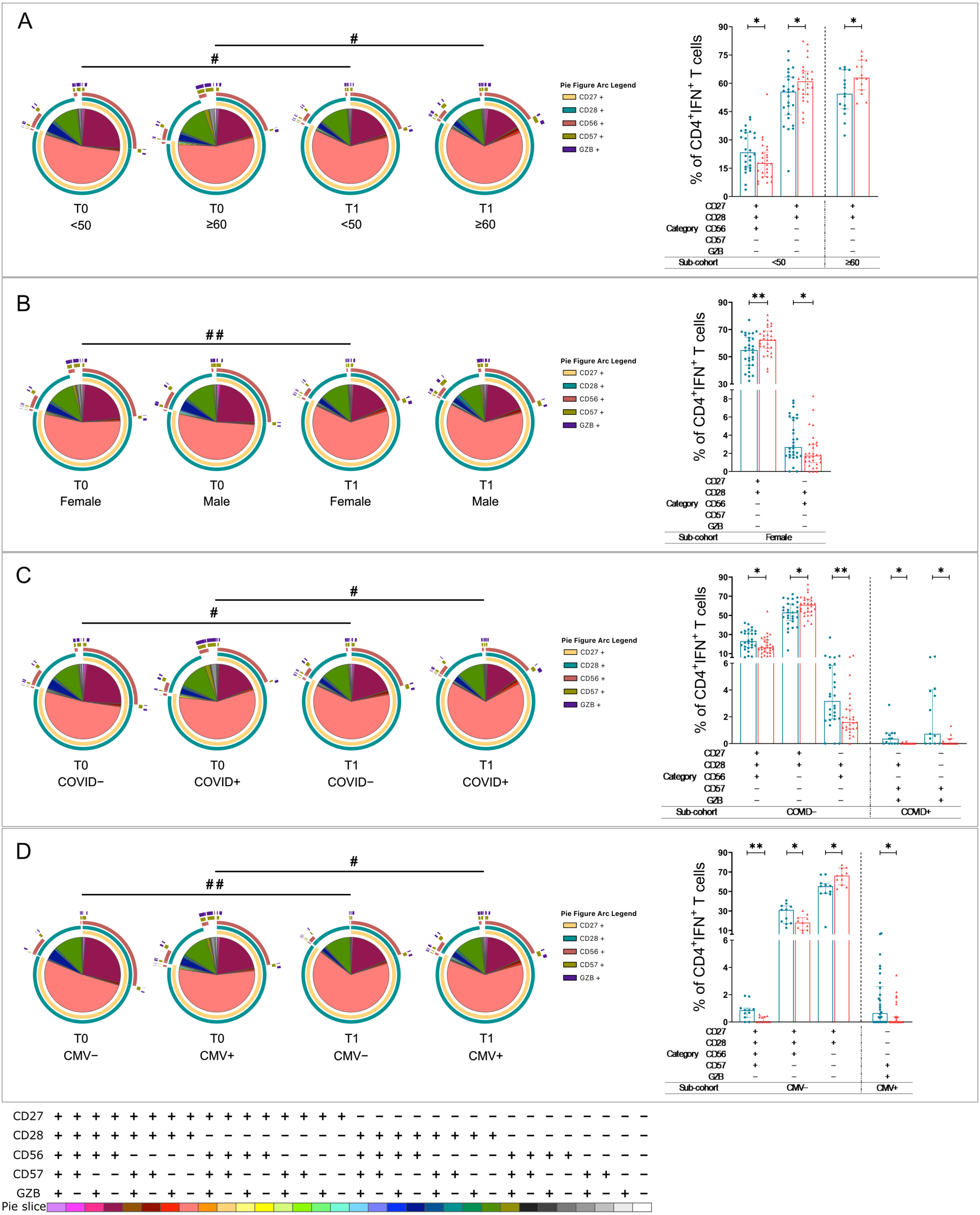

Figure S5. CD4+IFN-γ+ T-cell phenotype before (T0) and after (T1) the booster dose across sub-cohorts. Pie charts depict the phenotypic profile of CD4+IFN-γ+ T cells in each sub-cohort: age (**A**), sex (**B**), COVID (**C**) and CMV (**D**) at T0 (pies 1 and 2) and T1 (pies 3 and 4). Each of the 32 slices represents one combination of the markers CD27, CD28, CD56, CD57 and GZB, with legends shown below the charts. Marker expression is indicated by a plus sign (+), and lack of expression by a minus sign (−). Pie arcs correspond to each marker individually; overlapping arcs indicate coexpression of markers. Significantly different combinations (slices) are represented in the scatter plots to the right side of each panel. Top of bars indicate medians and whiskers represent the interquartile range, ranging from the 25th to the 75th percentile. A hash sign (#) denotes significance by permutation test (#*p* < 0.05; ##*p* < 0.01), denoting differences of phenotypic profile, while asterisks (*) indicate significance by Wilcoxon signed-rank test (**p* < 0.05; ***p* < 0.01), indicating differences in individual combinations/slices.

Table S6. Sub-cohort comparisons of CD4+ IFN-γ+ T-cell phenotype between T0 and T1.

Category					T0-T1 comparaison (*p*-value)							
					AGE		SEX		COVID		CMV	
CD27	CD28	CD56	CD57	GZB	<50	≥60	Female	Male	COVID−	COVID+	CMV−	CMV+
+	+	+	+	+	0.5459	0.5069	0.8187	0.3088	0.5175	0.6261	0.7180	0.4965
+	+	+	+	−	0.5581	0.6632	0.9823	0.1580	0.6000	0.4267	**0.0078**	0.6152
+	+	+	−	+	0.323	0.2902	0.7845	0.6695	0.8058	0.5727	0.3088	0.5946
+	+	+	−	−	**0.0301**	0.1776	**0.0519**	0.1077	**0.0213**	0.2702	**0.0328**	0.1103
+	+	−	+	+	0.3797	0.1711	0.1137	0.7180	0.1317	0.6629	0.1580	0.3555
+	+	−	+	−	0.9708	0.0779	0.6048	0.2244	0.6405	0.2090	0.9215	0.2009
+	+	−	−	+	0.2682	0.6632	0.3516	0.3764	0.2041	0.5903	0.4502	0.2838
+	+	−	−	−	**0.0481**	**0.0136**	**0.0097**	0.1783	**0.0209**	0.0612	**0.0138**	**0.0510**
+	−	+	+	+	0.5830	0.4937	0.7788	0.2786	0.4412	0.7005	0.4701	0.5493
+	−	+	+	−	0.4868	0.5338	0.6898	0.7180	1.0000	0.7779	0.4701	0.8016
+	−	+	−	+	0.7837	0.2134	0.3366	0.7180	0.3175	0.7389	0.7180	0.3366
+	−	+	−	−	0.9635	0.1013	0.5642	0.2786	0.8957	0.1062	0.6224	0.1558
+	−	−	+	+	0.2271	0.9835	0.2612	0.9738	0.4810	0.4571	0.4701	0.4688
+	−	−	+	−	0.6805	0.7244	0.6681	0.7180	0.8058	0.5050	0.7180	0.6574
+	−	−	−	+	0.7978	0.5338	0.6414	0.7180	0.4709	1.0000	0.7180	0.6414
+	−	−	−	−	0.7280	0.1985	0.6574	0.8955	0.6641	0.1585	0.2505	0.2838
−	+	+	+	+	0.7418	0.3095	0.8016	0.3410	0.4810	0.7389	0.7180	0.8016
−	+	+	+	−	0.6020	0.5476	0.8245	0.7180	0.9087	0.7779	0.9476	0.9882
−	+	+	−	+	0.4925	0.3507	0.7117	0.1486	0.2348	0.7389	0.4502	0.3711
−	+	+	−	−	0.0594	0.2134	**0.0195**	0.6695	**0.0058**	0.8576	0.1007	0.1120
−	+	−	+	+	0.9416	0.9339	0.6048	0.6224	0.2130	**0.0210**	0.9738	0.9117
−	+	−	+	−	0.1482	0.4186	0.1691	0.2786	0.5281	0.9795	0.4118	0.9528
−	+	−	−	+	0.8405	0.7557	0.7007	0.9215	0.5774	0.1178	0.6224	0.4508
−	+	−	−	−	0.2342	0.5755	0.4688	0.9738	0.6346	0.9387	0.6224	0.8360
−	−	+	+	+	0.6083	0.3297	0.3555	0.8182	0.3942	0.6816	0.6695	0.3366
−	−	+	+	−	0.4754	0.7557	0.6735	1.0000	0.6464	0.9795	1.0000	0.6735
−	−	+	−	+	0.3098	0.5897	0.2311	0.2786	0.6172	0.9591	1.0000	0.5692
−	−	+	−	−	0.6083	0.2717	0.4077	0.4701	0.2446	0.9387	0.9738	0.2311
−	−	−	+	+	0.1876	0.1198	0.0760	0.4502	0.3762	**0.0257**	0.9476	**0.0207**
−	−	−	+	−	0.4530	0.7244	0.8941	0.5327	0.6446	1.0000	0.4701	0.9764
−	−	−	−	+	0.8692	0.6187	0.2612	0.1486	0.8249	0.9387	0.9738	0.8245
−	−	−	−	−	0.4586	0.1198	0.3750	0.3410	0.7806	0.7389	0.3410	0.3671

Note. Statistical significance (*p*-values) is shown for the comparison between T0 and T1 within each sub-cohort (Wilcoxon signed-rank test). T0 = before boost; T1 = after boost.

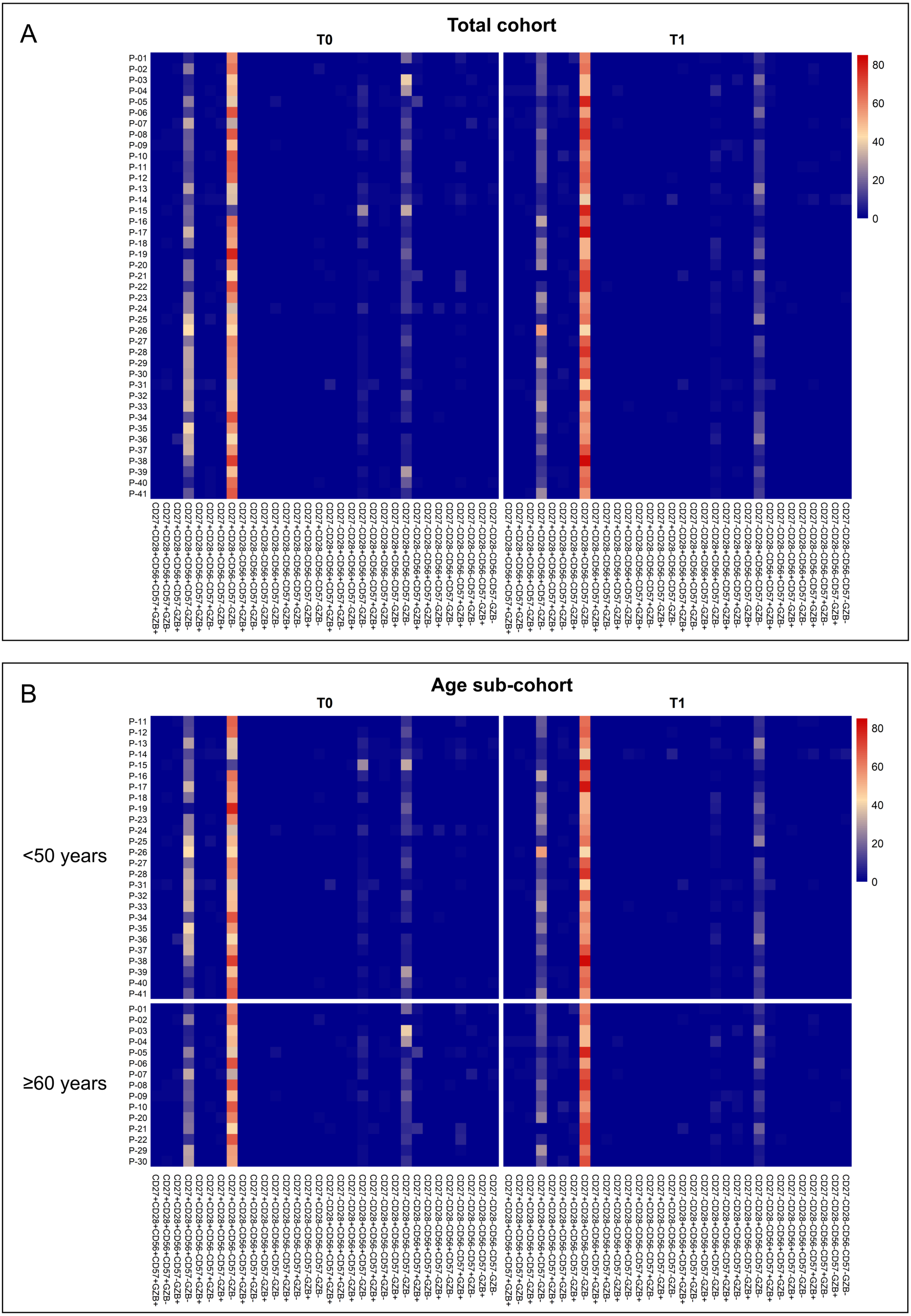

A
Total cohort
T0
T1
P-01
P-02
P-03
P-04
P-05
P-06
P-07
P-08
P-09
P-10
P-11
P-12
P-13
P-14
P-15
P-16
P-17
P-18
P-19
P-20
P-21
P-22
P-23
P-24
P-25
P-26
P-27
P-28
P-29
P-30
P-31
P-32
P-33
P-34
P-35
P-36
P-37
P-38
P-39
P-40
P-41
CD27+CD28+CD56+CD57+GZB+
CD27+CD28+CD56+CD57+GZB-
CD27+CD28+CD56+CD57-GZB+
CD27+CD28+CD56+CD57-GZB-
CD27+CD28+CD56-CD57+GZB+
CD27+CD28+CD56-CD57+GZB-
CD27+CD28+CD56-CD57-GZB+
CD27+CD28+CD56-CD57-GZB-
CD27+CD28-CD56+CD57+GZB+
CD27+CD28-CD56+CD57+GZB-
CD27+CD28-CD56+CD57-GZB+
CD27+CD28-CD56+CD57-GZB-
CD27+CD28-CD56-CD57+GZB+
CD27+CD28-CD56-CD57+GZB-
CD27+CD28-CD56-CD57-GZB+
CD27+CD28-CD56-CD57-GZB-
CD27-CD28+CD56+CD57+GZB+
CD27-CD28+CD56+CD57+GZB-
CD27-CD28+CD56+CD57-GZB+
CD27-CD28+CD56+CD57-GZB-
CD27-CD28+CD56-CD57+GZB+
CD27-CD28+CD56-CD57+GZB-
CD27-CD28+CD56-CD57-GZB+
CD27-CD28+CD56-CD57-GZB-
CD27-CD28-CD56+CD57+GZB+
CD27-CD28-CD56+CD57+GZB-
CD27-CD28-CD56+CD57-GZB+
CD27-CD28-CD56+CD57-GZB-
CD27-CD28-CD56-CD57+GZB+
CD27-CD28-CD56-CD57+GZB-
CD27-CD28-CD56-CD57-GZB+
CD27-CD28-CD56-CD57-GZB-
80
60
40
20
0
B
Age sub-cohort
T0
T1
<50 years
P-11
P-12
P-13
P-14
P-15
P-16
P-17
P-18
P-19
P-23
P-24
P-25
P-26
P-27
P-28
P-31
P-32
P-33
P-34
P-35
P-36
P-37
P-38
P-39
P-40
P-41
≥60 years
P-01
P-02
P-03
P-04
P-05
P-06
P-07
P-08
P-09
P-10
P-20
P-21
P-22
P-29
P-30
CD27+CD28+CD56+CD57+GZB+
CD27+CD28+CD56+CD57+GZB-
CD27+CD28+CD56+CD57-GZB+
CD27+CD28+CD56+CD57-GZB-
CD27+CD28+CD56-CD57+GZB+
CD27+CD28+CD56-CD57+GZB-
CD27+CD28+CD56-CD57-GZB+
CD27+CD28+CD56-CD57-GZB-
CD27+CD28-CD56+CD57+GZB+
CD27+CD28-CD56+CD57+GZB-
CD27+CD28-CD56+CD57-GZB+
CD27+CD28-CD56+CD57-GZB-
CD27+CD28-CD56-CD57+GZB+
CD27+CD28-CD56-CD57+GZB-
CD27+CD28-CD56-CD57-GZB+
CD27+CD28-CD56-CD57-GZB-
CD27-CD28+CD56+CD57+GZB+
CD27-CD28+CD56+CD57+GZB-
CD27-CD28+CD56+CD57-GZB+
CD27-CD28+CD56+CD57-GZB-
CD27-CD28+CD56-CD57+GZB+
CD27-CD28+CD56-CD57+GZB-
CD27-CD28+CD56-CD57-GZB+
CD27-CD28+CD56-CD57-GZB-
CD27-CD28-CD56+CD57+GZB+
CD27-CD28-CD56+CD57+GZB-
CD27-CD28-CD56+CD57-GZB+
CD27-CD28-CD56+CD57-GZB-
CD27-CD28-CD56-CD57+GZB+
CD27-CD28-CD56-CD57+GZB-
CD27-CD28-CD56-CD57-GZB+
CD27-CD28-CD56-CD57-GZB-
80
60
40
20
0

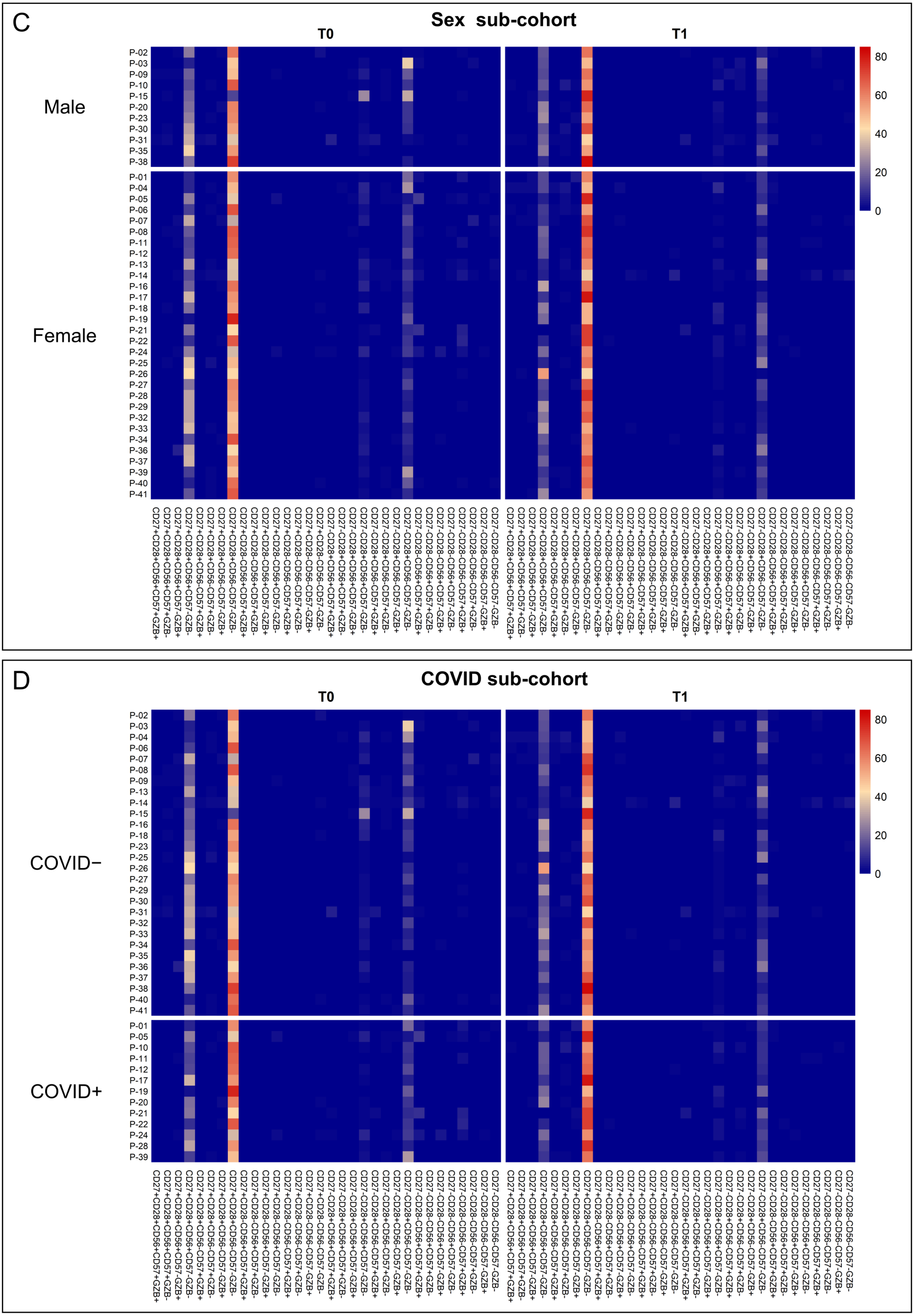
C
Sex sub-cohort
T0
T1
Male
Female
P-02
P-03
P-09
P-10
P-15
P-20
P-23
P-30
P-31
P-35
P-38
P-01
P-04
P-05
P-06
P-07
P-08
P-11
P-12
P-13
P-14
P-16
P-17
P-18
P-19
P-21
P-22
P-24
P-25
P-26
P-27
P-28
P-29
P-32
P-33
P-34
P-36
P-37
P-39
P-40
P-41
80
60
40
20
0
CD27+CD28+CD56+CD57+GZB+
CD27+CD28+CD56+CD57+GZB-
CD27+CD28+CD56+CD57-GZB+
CD27+CD28+CD56+CD57-GZB-
CD27+CD28+CD56-CD57+GZB+
CD27+CD28+CD56-CD57+GZB-
CD27+CD28+CD56-CD57-GZB+
CD27+CD28+CD56-CD57-GZB-
CD27+CD28-CD56+CD57+GZB+
CD27+CD28-CD56+CD57+GZB-
CD27+CD28-CD56+CD57-GZB+
CD27+CD28-CD56+CD57-GZB-
CD27+CD28-CD56-CD57+GZB+
CD27+CD28-CD56-CD57+GZB-
CD27+CD28-CD56-CD57-GZB+
CD27+CD28-CD56-CD57-GZB-
CD27-CD28+CD56+CD57+GZB+
CD27-CD28+CD56+CD57+GZB-
CD27-CD28+CD56+CD57-GZB+
CD27-CD28+CD56+CD57-GZB-
CD27-CD28+CD56-CD57+GZB+
CD27-CD28+CD56-CD57+GZB-
CD27-CD28+CD56-CD57-GZB+
CD27-CD28+CD56-CD57-GZB-
CD27-CD28-CD56+CD57+GZB+
CD27-CD28-CD56+CD57+GZB-
CD27-CD28-CD56+CD57-GZB+
CD27-CD28-CD56+CD57-GZB-
CD27-CD28-CD56-CD57+GZB+
CD27-CD28-CD56-CD57+GZB-
CD27-CD28-CD56-CD57-GZB+
CD27-CD28-CD56-CD57-GZB-
CD27+CD28+CD56+CD57+GZB+
CD27+CD28+CD56+CD57+GZB-
CD27+CD28+CD56+CD57-GZB+
CD27+CD28+CD56+CD57-GZB-
CD27+CD28+CD56-CD57+GZB+
CD27+CD28+CD56-CD57+GZB-
CD27+CD28+CD56-CD57-GZB+
CD27+CD28+CD56-CD57-GZB-
CD27+CD28-CD56+CD57+GZB+
CD27+CD28-CD56+CD57+GZB-
CD27+CD28-CD56+CD57-GZB+
CD27+CD28-CD56+CD57-GZB-
CD27+CD28-CD56-CD57+GZB+
CD27+CD28-CD56-CD57+GZB-
CD27+CD28-CD56-CD57-GZB+
CD27+CD28-CD56-CD57-GZB-
CD27-CD28+CD56+CD57+GZB+
CD27-CD28+CD56+CD57+GZB-
CD27-CD28+CD56+CD57-GZB+
CD27-CD28+CD56+CD57-GZB-
CD27-CD28+CD56-CD57+GZB+
CD27-CD28+CD56-CD57+GZB-
CD27-CD28+CD56-CD57-GZB+
CD27-CD28+CD56-CD57-GZB-
CD27-CD28-CD56+CD57+GZB+
CD27-CD28-CD56+CD57+GZB-
CD27-CD28-CD56+CD57-GZB+
CD27-CD28-CD56+CD57-GZB-
CD27-CD28-CD56-CD57+GZB+
CD27-CD28-CD56-CD57+GZB-
CD27-CD28-CD56-CD57-GZB+
CD27-CD28-CD56-CD57-GZB-
D
COVID sub-cohort
T0
T1
COVID−
COVID+
P-02
P-03
P-04
P-06
P-07
P-08
P-09
P-13
P-14
P-15
P-16
P-18
P-23
P-25
P-26
P-27
P-29
P-30
P-31
P-32
P-33
P-34
P-35
P-36
P-37
P-38
P-40
P-41
P-01
P-05
P-10
P-11
P-12
P-17
P-19
P-20
P-21
P-22
P-24
P-28
P-39
80
60
40
20
0
CD27+CD28+CD56+CD57+GZB+
CD27+CD28+CD56+CD57+GZB-
CD27+CD28+CD56+CD57-GZB+
CD27+CD28+CD56+CD57-GZB-
CD27+CD28+CD56-CD57+GZB+
CD27+CD28+CD56-CD57+GZB-
CD27+CD28+CD56-CD57-GZB+
CD27+CD28+CD56-CD57-GZB-
CD27+CD28-CD56+CD57+GZB+
CD27+CD28-CD56+CD57+GZB-
CD27+CD28-CD56+CD57-GZB+
CD27+CD28-CD56+CD57-GZB-
CD27+CD28-CD56-CD57+GZB+
CD27+CD28-CD56-CD57+GZB-
CD27+CD28-CD56-CD57-GZB+
CD27+CD28-CD56-CD57-GZB-
CD27-CD28+CD56+CD57+GZB+
CD27-CD28+CD56+CD57+GZB-
CD27-CD28+CD56+CD57-GZB+
CD27-CD28+CD56+CD57-GZB-
CD27-CD28+CD56-CD57+GZB+
CD27-CD28+CD56-CD57+GZB-
CD27-CD28+CD56-CD57-GZB+
CD27-CD28+CD56-CD57-GZB-
CD27-CD28-CD56+CD57+GZB+
CD27-CD28-CD56+CD57+GZB-
CD27-CD28-CD56+CD57-GZB+
CD27-CD28-CD56+CD57-GZB-
CD27-CD28-CD56-CD57+GZB+
CD27-CD28-CD56-CD57+GZB-
CD27-CD28-CD56-CD57-GZB+
CD27-CD28-CD56-CD57-GZB-
CD27+CD28+CD56+CD57+GZB+
CD27+CD28+CD56+CD57+GZB-
CD27+CD28+CD56+CD57-GZB+
CD27+CD28+CD56+CD57-GZB-
CD27+CD28+CD56-CD57+GZB+
CD27+CD28+CD56-CD57+GZB-
CD27+CD28+CD56-CD57-GZB+
CD27+CD28+CD56-CD57-GZB-
CD27+CD28-CD56+CD57+GZB+
CD27+CD28-CD56+CD57+GZB-
CD27+CD28-CD56+CD57-GZB+
CD27+CD28-CD56+CD57-GZB-
CD27+CD28-CD56-CD57+GZB+
CD27+CD28-CD56-CD57+GZB-
CD27+CD28-CD56-CD57-GZB+
CD27+CD28-CD56-CD57-GZB-
CD27-CD28+CD56+CD57+GZB+
CD27-CD28+CD56+CD57+GZB-
CD27-CD28+CD56+CD57-GZB+
CD27-CD28+CD56+CD57-GZB-
CD27-CD28+CD56-CD57+GZB+
CD27-CD28+CD56-CD57+GZB-
CD27-CD28+CD56-CD57-GZB+
CD27-CD28+CD56-CD57-GZB-
CD27-CD28-CD56+CD57+GZB+
CD27-CD28-CD56+CD57+GZB-
CD27-CD28-CD56+CD57-GZB+
CD27-CD28-CD56+CD57-GZB-
CD27-CD28-CD56-CD57+GZB+
CD27-CD28-CD56-CD57+GZB-
CD27-CD28-CD56-CD57-GZB+
CD27-CD28-CD56-CD57-GZB-

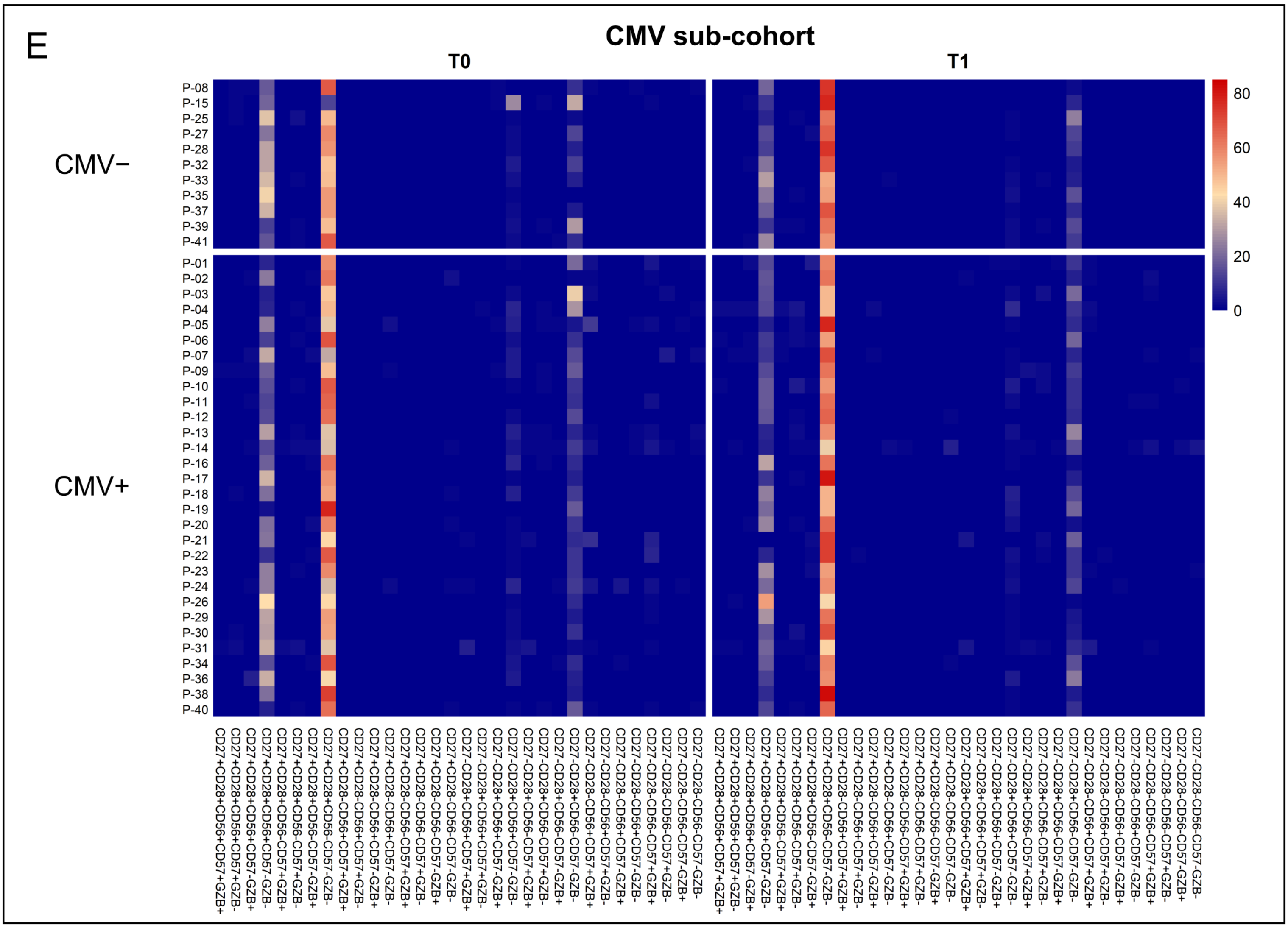

Figure S6. Heatmap representation of Boolean-derived phenotypic marker combinations for CD4+IFN-γ+ T cells. Percentages at T0 and T1 for each combination of the markers CD27, CD28, CD56, CD57 and GZB are shown for the total cohort (**A**) and for sub-cohort groups: age (**B**), sex (**C**), SARS-CoV-2 infection (COVID, **D**) and CMV serostatus (CMV, **E**). Participants are labelled from P-01 to P-41 along the left of each heatmap. T0 = before boost; T1= after boost.

Table S7. Partial correlations between humoral and CD8+ T-cell responses.

		**Humoral Response (HR),** anti-RBD IgG antibodies			
**CD8+ T-cell response (CR) readout**	**Statistics**	**ΔHR-ΔCR**	**T0 HR-T0 CR**	**T1 HR-T1 CR**	**T0 HR-T1 CR**
**% IFNt**	Correlation (*r*)	0,054	0,116	0,111	-0,006
	p-value	0,750	0,492	0,515	0,970
**% TNFt**	Correlation (*r*)	0,226	0,004	0,055	-0,048
	p-value	0,178	0,983	0,747	0,777
**iMFI IFNt**	Correlation (*r*)	0,322	0,061	0,298	0,048
	p-value	0,052	0,721	0,073	0,780
**iMFI TNFt**	Correlation (*r*)	0,203	0,117	-0,055	-0,138
	p-value	0,229	0,490	0,748	0,416
**% IFNm**	Correlation (*r*)	0,016	0,122	0,062	-0,029
	p-value	0,926	0,471	0,716	0,866
**% TNFm**	Correlation (*r*)	0,099	0,010	-0,284	-0,187
	p-value	0,561	0,952	0,089	0,268
**% bi**	Correlation (*r*)	0,324	-0,023	0,310	0,098
	p-value	0,050	0,894	0,062	0,564
**iMFI IFNm**	Correlation (*r*)	0,258	0,106	0,228	0,004
	p-value	0,123	0,531	0,174	0,982
**iMFI TNFm**	Correlation (*r*)	0,144	0,112	-0,273	-0,226
	p-value	0,395	0,510	0,102	0,178
**iMFI IFNbi**	Correlation (*r*)	**0,352***	-0,03	**0,352***	0,107
	p-value	0,033	0,860	0,033	0,528
**iMFI TNFbi**	Correlation (*r*)	0,289	0,051	0,264	0,061
	p-value	0,082	0,764	0,114	0,718

Note. Partial correlation coefficients (Pearson's *r*) and associated *p*-values were calculated between humoral response (HR, anti-RBD IgG antibodies) and CD8+ T-cell responses (CR), at T0, T1, or as increments (Δ) for each CD8+ functional readout listed in the left column of the table. Partial correlations were adjusted for sex, prior SARS-CoV-2 infection and CMV serostatus. Degrees of freedom = 35. Δ = increment; T0 = before boost; T1 = after boost; HR = Humoral Response; CR = Cellular Response. *p*-values are two-tailed. *$p < 0.05$.

Table S8. Linear models for clinical and demographic covariates effects on immune phenotypes.

	Type III SS ANOVA *p*-value	Estimated marginal mean		95% CI (lower limit–upper limit)	
**Immune phenotypes**	**AGE**	**<50**	**≥60**	**<50**	**≥60**
% of CD4+Treg	0,0341	5,04	6,37	4,09–5,99	5,2–7,54
% of CD8+CX3CR1+	0,0114	23,72	39,12	15,26–32,18	28,72–49,53
% of CD8+ TCM	0,0243	4,01	6,19	2,67–5,35	4,52–7,85
% of CD8+ TEMRA	0,0361	41,17	48,67	36,17–46,18	42,48–54,87
% of CD4+CD127+	0,0047	64,15	58,91	61,48–66,81	55,61–62,21
% of CD4+Treg TN	0,0229	19,73	14,65	16,62–22,84	10,8–18,49
% of CD8+ TN	0,0040	37,69	24,21	31,32–44,05	16,34–32,09
% of Total Monocytes	0,0105	2,63	3,59	2,12–3,15	2,95–4,22
% of NK	0,0106	4,85	7,55	3,38–6,32	5,75–9,36
% of NK CD56dimCD16+	0,0202	88,56	92,60	86,13–91	89,61–95,6
CD4:CD8 ratio	0,0326	2,43	3,43	1,73–3,13	2,57–4,29
	**SEX**	**Female**	**Male**	**Female**	**Male**
% of B cells	0,0145	3,78	4,93	3,23–4,33	4,08–5,78
	**CMV**	**CMV−**	**CMV+**	**CMV−**	**CMV+**
% of CD4+CD28−CD57+	0,0208	0,05	4,13	-3,12–3,22	2,34–5,91
% of CD8+CD28−CD57+	0,0042	14,12	29,77	3,4–24,83	23,75–35,79
% of NK CD56dimCD57+NKG2C+	0,0088	-3,74	9,60	-13,09–5,61	4,32–14,87
% of CD4+CX3CR1+	0,0217	1,76	7,30	-2,71–6,24	4,77–9,82
CD4:CD8 ratio	0,0102	3,58	2,28	2,58–4,58	1,72–2,84
	**COVID**	**COVID−**	**COVID+**	**COVID−**	**COVID+**
% of CD4+TEMRA	0,0003	4,88	9,75	3,35–6,42	7,42–12,07
% of CD4+Treg TEMRA	0,0000	8,16	13,05	6,83–9,5	11,03–15,07
% of CD8+ TCM	0,0150	3,93	6,27	2,76–5,1	4,49–8,04
% of CD4+Treg	0,0080	6,65	4,75	5,83–7,48	3,5–6,01
% of CD4+CD25+	0,0000	46,16	33,37	42,95–49,36	28,52–38,22
% of CD4+TCM	0,0076	37,22	29,18	33,57–40,86	23,66–34,71

Note. Linear models (ANCOVA) were used to assess the effects of the covariates age, sex, CMV serostatus (CMV) and prior SARS-CoV-2 infection (COVID) on T and innate cell phenotypes before vaccination. Only p-values < 0.05 are shown and they indicate a statistically significant effect of the covariate on the immune phenotype listed on the left side of the table. The estimated marginal means (EMMs) of the outcome values for each sub-cohort group are presented with corresponding lower and upper limits of a 95% confidence interval (CI). For all results, see **Table S5**. Negative EMMs arise from the statistical model fitting process. Values below 0 should be interpreted as very low or near-zero expression, as they do not represent biologically plausible values. SS = sums of squares.

Table S9. Partial correlations between immune phenotypes and anti-RBD IgG titres.

		**Humoral Response,** anti-RBD IgG		
**Phenotype of T cells**	**Statistics**	**T0**	**T1**	**Δ**
CD4:CD8 ratio	Correlation (*r*)	-0.155	-0.212	-0.142
	p-value	0.366	0.215	0.407
% of CD4+CD25+	Correlation (*r*)	-0.268	-0.282	-0.14
	p-value	0.115	0.095	0.417
% of CD4+CD127+	Correlation (*r*)	0.157	0.124	0.029
	p-value	0.361	0.47	0.866
% of CD4+CX3CR1+	Correlation (*r*)	0.173	0.103	-0.011
	p-value	0.314	0.548	0.949
% of CD4+CD28+CD57+	Correlation (*r*)	0.165	0.148	0.052
	p-value	0.336	0.389	0.762
% of CD4+CD28-CD57+	Correlation (*r*)	0.168	0.211	0.131
	p-value	0.328	0.216	0.445
% of CD4+TCM	Correlation (*r*)	0.038	-0.073	-0.125
	p-value	0.826	0.673	0.467
% of CD4+TEMRA	Correlation (*r*)	0.119	0.195	0.151
	p-value	0.49	0.255	0.378
% of CD4+TEMRA CD27-CD28+	Correlation (*r*)	-0.082	-0.069	-0.02
	p-value	0.635	0.69	0.907
% of CD4+TN	Correlation (*r*)	-0.131	0.005	0.116
	p-value	0.448	0.975	0.502
% of CD4+TN CD27-CD28+	Correlation (*r*)	-0.026	-0.115	-0.125
	p-value	0.88	0.506	0.466
% of CD4+Treg	Correlation (*r*)	0.024	0.25	0.301
	p-value	0.888	0.142	0.075
% of CD4+Treg TCM	Correlation (*r*)	-0.087	-0.092	-0.046
	p-value	0.615	0.594	0.791
% of CD4+Treg TEMRA	Correlation (*r*)	0.057	0.242	0.264
	p-value	0.743	0.155	0.12
% of CD4+Treg TN	Correlation (*r*)	0.125	0.224	0.184
	p-value	0.468	0.189	0.283
% of CD8+CD25+	Correlation (*r*)	0.087	0.257	0.257
	p-value	0.612	0.13	0.13
% of CD8+CD56+	Correlation (*r*)	0.263	0.051	-0.153
	p-value	0.121	0.767	0.372
% of CD8+CX3CR1+	Correlation (*r*)	0.194	-0.123	-0.32
	p-value	0.256	0.474	0.057
% of CD8+CD28-CD57+	Correlation (*r*)	0.227	-0.049	-0.252
	p-value	0.184	0.775	0.138
% of CD8+CD28+CD57+	Correlation (*r*)	-0.03	-0.174	-0.198
	p-value	0.861	0.31	0.247
% of CD8+ TCM	Correlation (*r*)	-0.054	-0.045	-0.012
	p-value	0.755	0.796	0.943
% of CD8+ TEM	Correlation (*r*)	-0.041	-0.213	-0.239
	p-value	0.811	0.213	0.161
% of CD8+ TEMRA	Correlation (*r*)	0.2	0.107	-0.028
	p-value	0.243	0.533	0.869

		Humoral Response, anti-RBD IgG		
**Phenotype of T cells**	**Statistics**	**T0**	**T1**	**Δ**
% of CD8+ TN	Correlation (*r*)	-0.11	0.108	0.231
	p-value	0.522	0.529	0.175
**Phenotype of innate cells**				
% of Lymphocytes	Correlation (*r*)	-0.02	-0.071	-0.074
	p-value	0.907	0.682	0.668
% of T cells	Correlation (*r*)	-0.043	-0.059	-0.04
	p-value	0.804	0.733	0.818
% of Total Monocytes	Correlation (*r*)	0.052	-0.08	-0.146
	p-value	0.764	0.644	0.397
% of Total Monocytes HLADR+	Correlation (*r*)	0.319	0.173	-0.044
	p-value	0.058	0.314	0.799
% of MoNoClas	Correlation (*r*)	-0.164	-0.276	-0.218
	p-value	0.339	0.103	0.201
% of MoNoClas HLADR+	Correlation (*r*)	0.086	-0.02	-0.097
	p-value	0.616	0.91	0.573
% of MoClas	Correlation (*r*)	0.052	0.175	0.181
	p-value	0.763	0.309	0.292
% of MoClas HLADR+	Correlation (*r*)	0.328	0.196	-0.022
	p-value	0.05	0.252	0.9
% of MoInter	Correlation (*r*)	-0.025	-0.153	-0.175
	p-value	0.883	0.373	0.307
% of MoInter HLADR+	Correlation (*r*)	0.294	0.217	0.033
	p-value	0.082	0.204	0.848
% of B cells	Correlation (*r*)	-0.128	-0.152	-0.088
	p-value	0.457	0.376	0.608
% of NK	Correlation (*r*)	0.109	-0.011	-0.105
	p-value	0.526	0.95	0.542
% of NK CD56br	Correlation (*r*)	-0.114	0.101	0.224
	p-value	0.51	0.557	0.188
% of NK CD56brCD57-NKG2C+	Correlation (*r*)	-0.274	-0.154	0.03
	p-value	0.106	0.37	0.86
% of NK CD56brCD57+NKG2C+	Correlation (*r*)	0.019	0.147	0.173
	p-value	0.912	0.393	0.314
% of NK CD56brCD57+NKG2C-	Correlation (*r*)	**0.349***	0.048	-0.229
	p-value	0.037	0.781	0.179
% of NK CD56brCD57-NKG2C-	Correlation (*r*)	0.173	0.121	0.012
	p-value	0.313	0.481	0.946
% of NK CD56dimCD16+	Correlation (*r*)	0.214	0.065	-0.095
	p-value	0.21	0.707	0.581
% of NK CD56dimCD57-NKG2C+	Correlation (*r*)	-0.295	-0.11	0.105
	p-value	0.08	0.523	0.544
% of NK CD56dimCD57+NKG2C+	Correlation (*r*)	0.016	-0.062	-0.093
	p-value	0.925	0.721	0.59
% of NK CD56dimCD57+NKG2C-	Correlation (*r*)	0.057	-0.008	-0.057
	p-value	0.743	0.965	0.741
% of NK CD56dimCD57-NKG2C-	Correlation (*r*)	-0.001	0.084	0.108
	p-value	0.997	0.628	0.531

		Humoral Response, anti-RBD IgG		
**Phenotype of innate cells**	**Statistics**	**T0**	**T1**	**∆**
% of Basophils	Correlation (*r*)	-0.044	0.048	0.098
	p-value	0.8	0.782	0.57
% of DCs	Correlation (*r*)	0.118	-0.081	-0.203
	p-value	0.494	0.637	0.236
% of mDCs	Correlation (*r*)	0.135	0.091	0.005
	p-value	0.433	0.596	0.977
% of nDCs	Correlation (*r*)	-0.049	0.004	0.046
	p-value	0.776	0.981	0.79
% of pDCs	Correlation (*r*)	-0.135	-0.119	-0.04
	p-value	0.433	0.491	0.817

Note. Partial correlation coefficients (Pearson's *r*) and associated *p*-values were calculated between the humoral response at T0, T1 or as increment (∆), and the phenotype of T and innate cells at T0 listed in the left column of the table. Partial correlations were adjusted for sex, prior SARS-CoV-2 infection and CMV serostatus. Degrees of freedom = 34. T0 = before boost; T1 = after boost; ∆ = increment of antibodies. *p*-values are two-tailed. **p* < 0.05.

Table S10. Partial correlations between CD8+ T-cell phenotype and CD8+ T-cell response at T1.

		**T0 CD8+ T-cell phenotype (%)**		
**T1 CD8+ T-cell response**	**Statistics**	**CD56+**	**CD28nullCD57+**	**CX3CR1+**
**% of IFNt**	Correlation (*r*)	-0.129	**-0.327***	**-0.347***
	p-value	0.446	0.048	0.035
**% of TNFt**	Correlation (*r*)	-0.124	-0.274	-0.288
	p-value	0.465	0.101	0.084
**iMFI of IFNt**	Correlation (*r*)	**-0.340***	**-0.348***	**-0.409***
	p-value	0.04	0.035	0.012
**iMFI of TNFt**	Correlation (*r*)	-0.148	-0.303	-0.284
	p-value	0.381	0.068	0.089
**% of IFNm**	Correlation (*r*)	-0.089	-0.318	-0.324
	p-value	0.599	0.055	0.051
**% of TNFm**	Correlation (*r*)	0.15	-0.058	0.016
	p-value	0.375	0.731	0.924
**% of bi cells**	Correlation (*r*)	-0.284	-0.296	**-0.376***
	p-value	0.089	0.076	0.022
**iMFI of IFNm**	Correlation (*r*)	-0.285	-0.314	**-0.365***
	p-value	0.087	0.058	0.026
**iMFI of TNFm**	Correlation (*r*)	0.052	-0.148	-0.066
	p-value	0.759	0.383	0.696
**iMFI of IFNbi**	Correlation (*r*)	**-0.363***	**-0.338***	**-0.405***
	p-value	0.027	0.041	0.013
**iMFI of TNFbi**	Correlation (*r*)	-0.322	**-0.325***	**-0.399***
	p-value	0.052	0.05	0.015

Note. Partial correlation coefficients (Pearson's *r*) and associated *p*-values were calculated between the CD8+ T-cell phenotype at T0 and the cellular response of CD8+ T cells at T1 listed in the left column of the table. Partial correlations were adjusted for sex, prior SARS-CoV-2 infection and CMV serostatus. Degrees of freedom = 34. T0 = before boost; T1 = after boost; ∆ = increment of antibodies. *p*-values are two-tailed. **p* < 0.05.

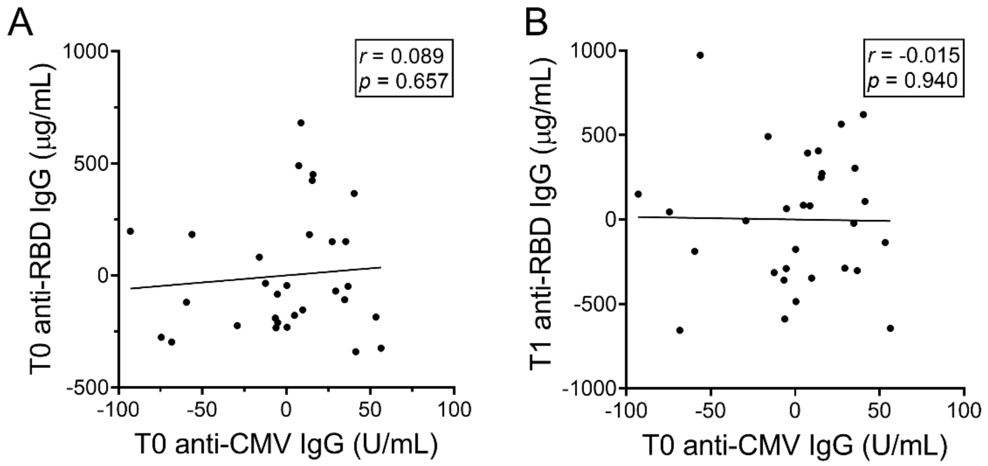

Figure S7. **Partial correlations between anti-CMV IgG and anti-RBD IgG levels among CMV+ individuals**. Correlations (Pearson's *r*) were assessed between anti-CMV IgG levels at T0 and SARS-CoV-2 anti-RBD IgG levels at T0 (**A**) and T1 (**B**), using residuals derived from linear regression models adjusted for sex, age, and prior SARS-CoV-2 infection. Degrees of freedom = 25. T0 = before boost; T1 = after boost.

Table S11. Partial correlations between anti-CMV IgG and T-cell responses at T0.

T0 T-cell response (CR) readout	Statistics	anti-CMV IgG-CD4+ CR	anti-CMV IgG-CD8+ CR
**% IFNt**	Correlation (*r*)	-0,203	0,086
	p-value	0,309	0,668
**% TNFt**	Correlation (*r*)	0,113	0,123
	p-value	0,573	0,54
**iMFI IFNt**	Correlation (*r*)	-0,146	-0,252
	p-value	0,467	0,205
**iMFI TNFt**	Correlation (*r*)	0,115	0,191
	p-value	0,567	0,339
**% IFNm**	Correlation (*r*)	-0,188	0,127
	p-value	0,347	0,526
**% TNFm**	Correlation (*r*)	0,225	0,237
	p-value	0,258	0,233
**% bi**	Correlation (*r*)	0	-0,383*
	p-value	0,315	0,049
**iMFI IFNm**	Correlation (*r*)	-0,221	-0,025
	p-value	0,267	0,900
**iMFI TNFm**	Correlation (*r*)	0,201	0,242
	p-value	0,314	0,224
**iMFI IFNbi**	Correlation (*r*)	-0,103	**-0,500****
	p-value	0,609	0,008
**iMFI TNFbi**	Correlation (*r*)	0	-0,412
	p-value	0,250	0,033

Note. Among CMV+ individuals, partial correlation coefficients (Pearson's *r*) and associated *p*-values were calculated between the levels of anti-CMV IgG at T0 and the cellular response readouts of CD4+ and CD8+ T cells at T0 listed in the left column of the table. Partial correlations were adjusted for sex, prior SARS-CoV-2 infection and CMV serostatus. Degrees of freedom = 25. T0 = before boost. *p*-values are two-tailed. ***p* < 0.01.

Table S12. Partial correlations between anti-CMV IgG and T-cell responses at T1.

T1 T-cell response (CR) readout	Statistics	anti-CMV IgG-CD4+ CR	anti-CMV IgG-CD8+ CR
**% IFNt**	Correlation (*r*)	**-0,487****	**-0,551****
	p-value	0,010	0,003
**% TNFt**	Correlation (*r*)	-0,207	-0,434
	p-value	0,301	0,024
**iMFI IFNt**	Correlation (*r*)	-0,354	-0,469
	p-value	0,070	0,014
**iMFI TNFt**	Correlation (*r*)	-0,194	-0,308
	p-value	0,332	0,119
**% IFNm**	Correlation (*r*)	-0,451	**-0,509****
	p-value	0,018	0,007
**% TNFm**	Correlation (*r*)	-0,054	-0,009
	p-value	0,788	0,965
**% bi**	Correlation (*r*)	-0,337	**-0,572****
	p-value	0,085	0,002
**iMFI IFNm**	Correlation (*r*)	-0,269	-0,339
	p-value	0,174	0,084
**iMFI TNFm**	Correlation (*r*)	-0,058	-0,018
	p-value	0,774	0,928
**iMFI IFNbi**	Correlation (*r*)	-0,384	**-0,559****
	p-value	0,048	0,002
**iMFI TNFbi**	Correlation (*r*)	-0,372	**-0,533****
	p-value	0,056	0,004

Note. Among CMV+ individuals, partial correlation coefficients (Pearson's *r*) and associated *p*-values were calculated between the levels of anti-CMV IgG at T0 and the cellular response readouts of CD4+ and CD8+ T cells at T1 listed in the left column of the table. Partial correlations were adjusted for sex, prior SARS-CoV-2 infection and CMV serostatus. Degrees of freedom = 25. T1 = after boost. *p*-values are two-tailed. **$p < 0.01$.
